# Toenail arsenic species and metallome profiles associated with breast, cervical, prostate, and skin cancer prevalence in the Atlantic Partnership for Tomorrow’s Health cohort

**DOI:** 10.3389/fpubh.2023.1148283

**Published:** 2023-06-15

**Authors:** Kalli M. Hood, Ellen Sweeney, Gabriela Ilie, Erin Keltie, Jong Sung Kim

**Affiliations:** ^1^Health and Environments Research Centre (HERC) Laboratory, Faculty of Medicine, Dalhousie University, Halifax, NS, Canada; ^2^Department of Community Health & Epidemiology, Faculty of Medicine, Dalhousie University, Halifax, NS, Canada; ^3^Atlantic Partnership for Tomorrow’s Health (PATH), Faculty of Medicine, Dalhousie University, Halifax, NS, Canada; ^4^Department of Occupational and Environmental Health, College of Public Health, The University of Iowa, Iowa City, IA, United States

**Keywords:** arsenic speciation, metallomics, metal exposure, biomarkers, toenails, cancer risk

## Abstract

**Introduction:**

Chronic exposure to arsenic through drinking water has been linked to several cancers. The metabolism of arsenic is thought to play a key role in arsenic-related carcinogenesis as metabolites of varying toxicity are produced and either stored in or excreted from the body. Atlantic Canada has the highest age-standardized incidence rates of all cancers in the country. This may be due to its high levels of environmental arsenic and the prevalence of unregulated private wells for water consumption. Here, we aimed to characterize the profiles of arsenic species and metallome in the toenails of four cancer groups, compare them to healthy participants (*N* = 338), and assess potential associations between the profiles with cancer prevalence.

**Methods:**

This study employed a case–control design. Toenail samples and questionnaire data from cases (breast, cervical, prostate, and skin cancers) and controls were sourced from the Atlantic Partnership for Tomorrow’s Health (PATH) cohort study. The levels of arsenic species were measured using Inductively Coupled Plasma-Mass Spectrometry (ICP-MS) paired with High Performance Liquid Chromatography (HPLC) and total concentrations of metallome (23 metals) were determined by ICP-MS separately. Multivariate analyses were conducted to compare cases with controls within each cancer group.

**Results:**

Arsenic speciation profiles varied by cancer type and were significantly different between cases and controls in the breast (*p* = 0.0330), cervical (*p* = 0.0228), and skin (*p* = 0.0228) cancer groups. In addition, the profiles of metallome (nine metals) were significantly differentiated in the prostate (*p* = 0.0244) and skin (*p* = 0.0321) cancer groups, with higher zinc concentrations among cases compared to controls.

**Conclusion:**

History of cancer diagnosis was associated with specific profiles of arsenic species and metallome. Our results indicate that arsenic methylation and zinc levels, as measured in toenails, may be an important biomarker for cancer prevalence. Further research is needed to use toenails as a prognostic measure of arsenic-and other metal-induced cancer.

## Graphical Abstract

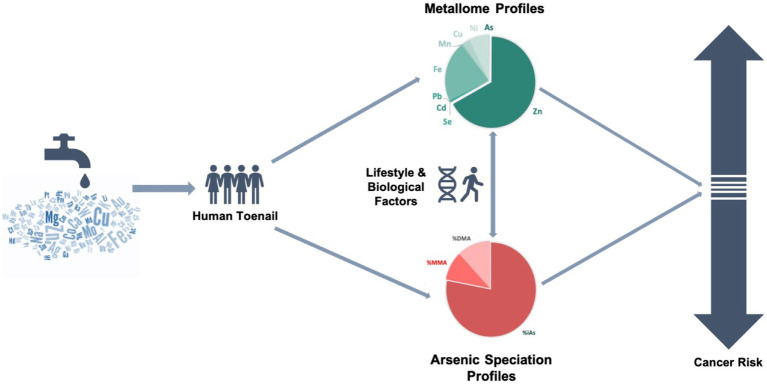

## 1. Introduction

Inorganic arsenic compounds have been classified as a Group 1 carcinogen by the International Agency for Research on Cancer (IARC) due to its association with several types of cancer, including lung, bladder, kidney, prostate, skin, and liver (1). The primary exposure pathway is *via* drinking water containing arsenic, where the inorganic form is predominant (2). In Atlantic Canada, high levels of arsenic in the groundwater have been linked with the bedrock formation (3, 4). Private well owners whose source water has high levels of environmental arsenic are at risk of arsenic exposure as exposure monitoring is not regulated. Drinking water containing arsenic has been shown to be a predominant source of arsenic body burden – the difference in amount of arsenic stored and excreted from the body – in this area (3, 5). At the same time, the Atlantic region has the highest age-standardized incidence rates of cancer in the country (6).

The mechanisms of arsenic-induced cancers have yet to be fully elucidated; however, the process of arsenic methylation (alternative reduction and addition of a methyl group (7)) has already emerged as a potential key mediator (7–11). Specifically, the methylation of inorganic arsenic (iAs) produces two metabolites: monomethylarsonic acid (MMA) and dimethylarsinic acid (DMA) (7, 8). This process was once considered a detoxification pathway as some of the metabolites, MMA (V) and DMA (V), are less reactive with tissue and more readily excreted in urine; however, incomplete methylation may produce residual MMA (III) or DMA (III), which are more reactive and thus considered more toxic due to its inhibition of DNA repair, suppression of cell cycle checkpoint proteins, and oxidative stress, by disturbing the pro/antioxidant balance (7, 12). In fact, an *in vitro* study demonstrated that MMA (III) is the most toxic intermediate produced from arsenic metabolism (13). The extent of methylation capacity has been quantified using two measures: the primary methylation index (PMI) and secondary methylation index (SMI); the former is the ratio of the levels of MMA to iAs and the latter is the ratio of the levels of DMA to MMA as measured in the given biomarker. Arsenic methylation capacity varies at both the population and individual levels; influential factors include age, sex, body mass index (BMI), and genomic polymorphisms in the arsenic (III) methyltransferase enzyme (12, 14–16). While further research is warranted to better understand these variations, arsenic methylation capacity is widely considered to be important in understanding arsenic-related toxicity and carcinogenesis, which have been previously described (12, 17, 18).

Metallomics is an emerging topic of metallomes that refer to the entirety of metals and metalloid species among the ‘omics’ sciences, which includes genomics, proteomics, and metabolomics (19, 20). Metallomes are characterized given their essential roles in the expression of biological and physiological functions, metalloenzymes, and the toxic nature of some elements (20, 21). While the field can be applied across different biological and biochemical areas, the interrelationship of elements’ speciation and concentrations renders metallomics important in toxicological research and understanding the effects of trace metal exposure. Often categorized as essential or toxic, many trace metals have been associated with cancers, but findings have been conflicting across epidemiological studies (22). One possible explanation for this is a failure to comprehensively assess the metallome profiles and the scarcity of such evaluations, which represents a critical gap in evaluating the potential health effects given the interactions between metals (22, 23). To our knowledge, only two studies have used urinary metallomics to identify prognostic factors for pancreatic cancer (24) and breast cancer (25).

The hypothesized mechanisms of trace metal-induced carcinogenicity largely depend on dosage, oxidation states, and chemical structures. Some metals, including magnesium (Mg), copper (Cu), and zinc (Zn) are considered essential (24), and their deficiency is thought to lead to the loss of protective factors. For example, a nutritional deficiency of zinc has conventionally been thought to increase cancer risk as it plays an important role in the synthesis of metallothionine, which is thought to inhibit free radical production (26). However, the range of zinc concentrations and their association with cancer have been varied among existing observational studies (23, 27–29), and a reliable biomarker has yet to be established for assessing zinc levels and those of other metals (30). Recently, there has been a shift to acknowledging that the interrelationship between metals may play an important role in the development of cancer (22, 31). Lead (Pb) is a key example of this, where in addition to inhibited DNA repair and chromosomal aberrations, it has been reported to create synergistic effects with other metals and carcinogens (32). While the mechanisms of effect are less clear for some metals, others are widely considered toxic with no safe levels: both arsenic and cadmium (Cd) have been labeled carcinogenic due to their association with the activation of proto-oncogenes, inactivation of tumor suppressor genes, cell proliferation, inhibition of deoxyribonucleic acid (DNA) repair, and generation of reactive oxygen species (ROS) (31, 32).

There is increasing evidence to indicate that metal speciation plays an important role in toxicity, particularly in the case of arsenic (14, 33–35). The use of urine biomarkers to measure arsenic species is useful to investigate potential associations using known methodologies; however, the use of more advanced techniques and long-term biomarkers (i.e., metallome and speciation analysis with toenails) may further our understanding of the link between chronic heavy metal exposure, arsenic speciation, and cancer. Recently, there has been growing recognition for the use of toenails as biomarkers for metals exposure, and particularly arsenic given its affinity for the keratin-rich tissue, the long growth period, and limited external contamination (36). Moreover, arsenic concentrations in toenails are strongly correlated with drinking water concentrations (3, 36) and may be indicative of the body burden of arsenic.

This study expanded our previous work by characterizing the profiles of arsenic species and metallomes in human toenails from the Atlantic Partnership for Tomorrow’s Health (Atlantic PATH) cohort and focusing on less studied cancers (cervical, breast and prostate cancers), which have also been linked to arsenic exposure (33, 34). Specifically, we aimed to assess the relationship between the profiles of arsenic species and metallomes and cancers, and determine the roles of these profiles in cancer cases (37).

## 2. Materials and methods

### 2.1. Study design and population

**Figure 1:**
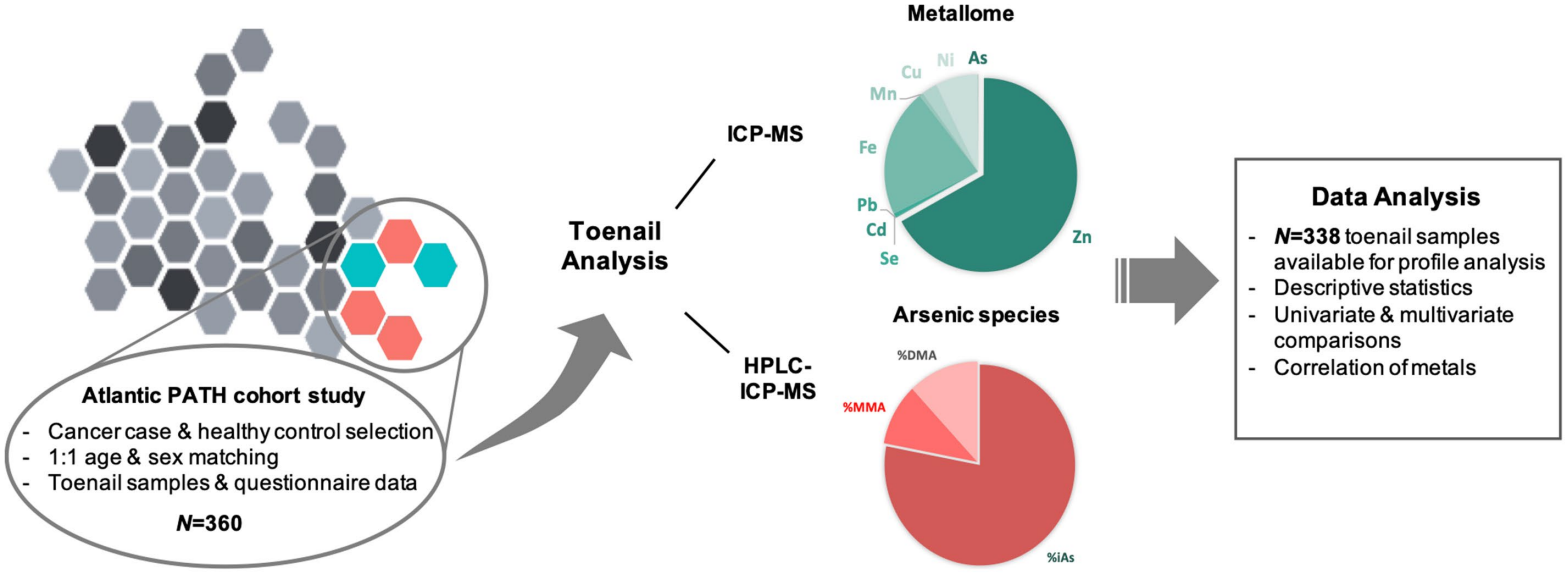
Research workplan overview.

This analytic sample consisted of 338 individuals (1:1 matched cases and controls) who participated in the baseline collection (2009–2015) of the Atlantic PATH study, which is part of the largest prospective cohort study in Canada, the Canadian Partnership for Tomorrow’s Health (CanPath, formerly the Canadian Partnership for Tomorrow Project). Data holdings included questionnaire data, physical measures and biosamples, which consisted of blood, urine, saliva, and toenail samples, collected both in person and at home. Details on the study population and data collection in the Atlantic PATH cohort is described in detail elsewhere (38, 39). This study used a case–control study design, matched on sex and age (+/−5 years) for each of the cancer types, individually. The cancer types of interest were breast, cervical, prostate, and skin cancer. Cancer cases were randomly selected for inclusion if participants had a history of the cancer of interest, but no other health conditions. Among the cases, there were 41 individuals with a history of breast cancer, 41 with a history of cervical cancer, 44 with a history of prostate cancer, and 43 with a history of skin cancer. The randomly selected healthy matched controls must have never had a history of cancer, diabetes, or cardiovascular disease. Participants’ toenail samples were analyzed for metallomes and arsenic species and compared between cases and controls (Figure 1).

### 2.2. Participant characteristics

Participant demographics and characteristics are provided in Table 1. The mean ages across the groups ranged from 50–60 years, and the skin cancer group was 60% women. Participants largely sourced their water from municipal sources (34–51%) or private wells (12–27%), had moderate to high levels of physical activity, were overweight or obese, and were not current smokers. Data collection and participant characteristics are described in greater detail in the Atlantic PATH Cohort Profile (38).

**Table 1:** Participant characteristics by case/control group; data sourced from the Atlantic PATH cohort study (*N* = 338).

	Breast cancer group (*n* = 82) count (%)		Cervical cancer group (*n* = 82) count (%)		Prostate cancer group (*n* = 88) count (%)		Skin cancer group (*n* = 86) Count (%)	
	Case	Control	Case	Control	Case	Control	Case	Control
Sex								
Female	41 (100%)	41 (100%)	41 (100%)	41 (100%)	–	–	26 *(60.5%)*	26 *(60.5%)*
Male	–	–	–	–	44 (100%)	44 (100%)	17 *(39.5%)*	17 *(39.5%)*
Age, mean *(SD)*	57.4 *(7.2)*	57.4 *(7.2)*	51.8 *(8.2)*	51.7 *(8.2)*	60.9 *(4.8)*	60.9 *(4.8)*	55.7 *(8.7)*	55.7 *(8.7)*
39–59	21 *(51%)*	21 *(51%)*	33 *(80%)*	33 *(80%)*	18 *(41%)*	18 *(41%)*	23 *(53%)*	23 *(53%)*
60–69	20 *(49%)*	20 *(49%)*	8 *(20%)*	8 *(20%)*	26 *(59%)*	26 *(59%)*	20 *(47%)*	20 *(47%)*
Family History of Cancer	29 *(71%)*	31*(76%)*	25 *(62.5%)*	32 *(78%)*	28 *(67%)*	29 *(69%)*	30 *(71%)*	29 *(67%)*
Province								
NB	17 *(42%)*	17 *(42%)*	16 *(39%)*	15 *(37%)*	19 *(43%)*	19 *(43%)*	14 *(33%)*	14 *(33%)*
NL	6 *(15%)*	6 *(15%)*	13 *(32%)*	13 *(32%)*	14 *(32%)*	14 *(32%)*	14 *(33%)*	14 *(33%)*
NS	14 *(34%)*	14 *(34%)*	11 *(27%)*	13 *(32%)*	10 *(23%)*	10 *(23%)*	11 *(26%)*	11 *(26%)*
PEI	4 *(10%)*	4 *(10%)*	1 *(2%)*	0 *(0%)*	2 *(2%)*	1 *(2%)*	4 *(9%)*	4 *(9%)*
Water Source								
Municipal	15 *(37%)*	14 *(34%)*	19 *(46%)*	18 *(44%)*	12 *(27%)*	18 *(41%)*	22 *(51%)*	17 *(39%)*
Well	10 *(24%)*	10 *(24%)*	9 *(22%)*	10 *(24%)*	12 *(27%)*	8*(18%)*	5 *(12%)*	11*(26%)*
Other	2 *(5%)*	1 *(2%)*	1 *(2%)*	3 *(7%)*	0 *(0%)*	1 *(2%)*	2 *(5%)*	2 *(5%)*
Missing	14 *(34%)*	16 *(39%)*	12 *(29%)*	10 *(24%)*	20 *(45%)*	17 *(39%)*	14 *(33%)*	13 *(30%)*
Physical Activity								
Low	11 *(27%)*	8 *(19%)*	5 *(12%)*	7 *(17%)*	*9 (20%)*	11 *(25%)*	*3 (7%)*	7 *(16%)*
Moderate	14 *(34%)*	15 *(37%)*	16 *(39%)*	15 *(37%)*	14 *(32%)*	8 *(18%)*	19 *(44%)*	19 *(44%)*
High	16 *(39%)*	18 *(44%)*	20 *(49%)*	19 *(46%)*	21 *(48%)*	25 *(57%)*	21 *(49%)*	17 *(40%)*
Smoking								
Never	27 *(66%)*	19 *(46%)*	16 *(39%)*	26 *(63%)*	21 *(48%)*	18 *(41%)*	24 *(56%)*	19 *(44%)*
Former	13 *(32%)*	20 *(49%)*	18 *(44%)*	12 *(29%)*	18 *(41%)*	23 *(52%)*	16 *(37%)*	20 *(47%)*
Current	1 *(2%)*	2 *(5%)*	7 *(17%)*	3 *(7%)*	5 *(11%)*	3 *(7%)*	3 *(7%)*	4 *(9%)*
BMI, mean *(SD)*	29.8 *(9.4)*	27.3 *(4.9)*	28.9 *(5.9)*	28.5 *(6.5)*	29.1 *(4.6)*	29.6 *(4.9)*	28.2 *(5.1)*	28.8 *(6.2)*
BMI Categories								
Low/Normal	9 *(22%)*	9 *(22%)*	7 *(17%)*	7 *(17%)*	4 *(9%)*	2 *(5%)*	11 *(26%)*	7 *(16%)*
Overweight	8 *(19%)*	11 *(27%)*	11 *(27%)*	10 *(24%)*	13 *(30%)*	16 *(36%)*	7 *(16%)*	13 *(30%)*
Obese	6 *(15%)*	8 *(19%)*	6 *(15%)*	*6 (15%)*	9 *(20%)*	11 *(25%)*	12 *(28%)*	*7 (16%)*
Unknown	18 *(44%)*	13 *(32%)*	17 *(41%)*	18 *(44%)*	18 *(41%)*	15 *(24%)*	13 *(30%)*	16 *(37%)*

### 2.3. Toenail sample preparation

The method used to digest and analyze toenails has been previously described and validated (33, 34). In brief, toenail samples were weighed to an approximate mass of 50 mg and transferred to a 10 mL quartz vial for cleaning. Toenails were submerged, sonicated, and rinsed with acetone, and this process was repeated using deionized water (Milli-Q Advantage A10 unit, MilliporeSigma, ON Canada). Cleaned samples were then dried in a Heratherm 60 L gravity convection oven (Thermo Scientific, MA USA) at 105°C overnight. Toenail samples were digested using a Discover SP-D microwave digestor (CEM Corporation, NC USA). In each sample vial 100 μL of concentrated nitric acid (HNO_3_), 500 μL of hydrogen peroxide (H_2_O_2_), and 400 μL of water were added. Three blanks were created with the same solution and analyzed alongside each batch. Digested samples were transferred to 15 mL polypropylene tubes and diluted tenfold with water; the final concentration of HNO_3_ was 1% (v/v). For the speciation analysis, a 995 μL aliquot of each sample and the blanks were transferred to 1.8 mL polypropylene vials (Thermo Scientific, MA United States). Finally, 5 μL of 20 μg/L arsenobetaine (AsB, Sigma Aldrich, MO United States) in 1% v/v HNO_3_ was added to each vial as an internal standard. AsB was chosen as internal standard as it represents a small fraction of total As found in toenails and is often undetectable (33, 40, 41). Samples were vortexed (Maxi Mix I, Thermo Scientific, MA United States) immediately before Inductively Coupled Plasma-Mass Spectrometry (ICP-MS) and High Performance Liquid Chromatography/ICP-MS (HPLC-ICP-MS) analysis to ensure homogeneity.

### 2.4. Total metals analysis by ICP-MS

Total concentration of arsenic and 22 other heavy metals (Li, V, Cr, Mn, Fe, Co, Ni, Cu, Zn, Ga, Se, Rb, Sr, Ag, Cd, Sb, Ba, Hg, Tl, Pb, Th, and U) in the toenail samples was measured using an ICP-MS (iCAP Q, Thermo Scientific, MA United States). An internal standard of 50 μg/L scandium (Sc, AccuStandard, CT USA) in 1% (v/v) HNO_3_ was added. All metallomes were analyzed in kinetic energy mode, with the exception of selenium (Se), which was measured in standard mode. High purity helium (>99.999% He) was used as the collision gas. The multi-element calibration standard was diluted to concentrations of 0.01, 0.05, 0.1, 0.5, 1, 5, 10, 50, and 100 μg/L in 1% (v/v) HNO_3_ to form a nine-point calibration curve. Quality control check standards (1 and 10 μg/L) were measured every 15 samples. Qtegra Intelligent Scientific Data Solution software (version 2.7, Thermo Scientific, MA United States) was used to collect and process data from total arsenic analysis.

### 2.5. Arsenic speciation analysis by HPLC/ICP-MS

To determine the levels of arsenic species, a separate analysis was performed using HPLC coupled with ICP-MS using a previously validated method (33). Briefly, the HPLC was fit with a P4000 pump, AS3000 autosampler, and SN4000 interface module. PEEK tubing was used to pair the HPLC with the ICP-MS. Sample injection and measurement was coordinated using the Qtregra Intelligent Scientific Data solution software. Arsenic species were separated with an anion exchange column (IonPac AS7, 250×2 mm, Thermo Scientific, MA, United States) and guard column (IonPac AG7, 50×2 mm, Thermo Scientific, MA, United States). Ammonium carbonate was used as the mobile phase using a gradient solution between 20 mM and 200 mM to achieve adequate separation for the arsenic species measured. A calibration stock solution was made by mixing individual arsenic (III), arsenic (V), MMA, and DMA standards. The stock solution was then diluted to 0.02, 0.05, 0.1, 0.2, 0.5, 1, and 2 μg/L in 1% (v/v) HNO_3_ to form a seven-point calibration curve.

The method was previously validated and published (33, 34), but is briefly described hereafter. To date, there is no certified reference material for trace metal concentrations or arsenic speciation in toenails. Validation of the analytical methods was performed instead using certified human hair and frozen urine. NIES No. 13 Human Hair (National Institute for Environmental Studies, Tsukuba, Japan) samples were prepared using the toenail protocol. The method was tested for different sample masses (as little as 5 mg), as not all toenail samples had sufficient mass (50 mg). NIST 2669 Arsenic Species in Frozen Urine were used to validate the arsenic speciation method.

Method detection limits (MDL) were calculated using United States Environmental Protection Agency procedures (EPA 821-R-16-006) and the method blanks that were analyzed alongside the toenail samples (Supplementary Table S1). Previous studies (42–45) have found that replacing values below MDL with MDL/√2 does not introduce bias when the percentage of values below MDL is below 25%. As such, values for arsenic species that fell below MDL were replaced with the MDL/√2 for that species before being normalized by sample mass.

### 2.6. Statistical analysis

After toenail analysis, the four cancer case–control groups were analyzed independently: breast cancer (*n* = 82), cervical cancer (*n* = 82), prostate cancer (n = 88), and skin cancer (*n* = 86). Stata 15 was used to generate descriptive statistics and analyze the data (46). Figures were generated in RStudio using the ggplot package (47, 48). Demographic and participant characteristics are described with frequencies and were compared with chi-squared tests. Arsenic species and total metallome were non-normally distributed, however, in line with the Central Limit Theorem, we can assume that for sample sizes greater than 15, the errors will be normally distributed. As such, student’s t-tests were used to compare total concentrations of nine metallomes of interest (As, Mn, Fe, Ni, Cu, Zn, Se, Cd, and Pb) and arsenic species between cases and controls in each cancer group. Multivariate analysis of variance (MANOVA) was used to compare the profiles of arsenic species (%MMA, %DMA, %iAs, PMI, SMI) between cases and controls in each group. The models were then replicated using multivariate analysis of covariance (MANCOVA) to determine whether the covariates were significant predictors of the profiles of arsenic species. The correlations of metallomes (18) for cases and controls in each group were evaluated. All statistical tests were evaluated with a significance level of α = 0.05.

## 3. Results

### 3.1. Arsenic species profiles

**Figure 2:**
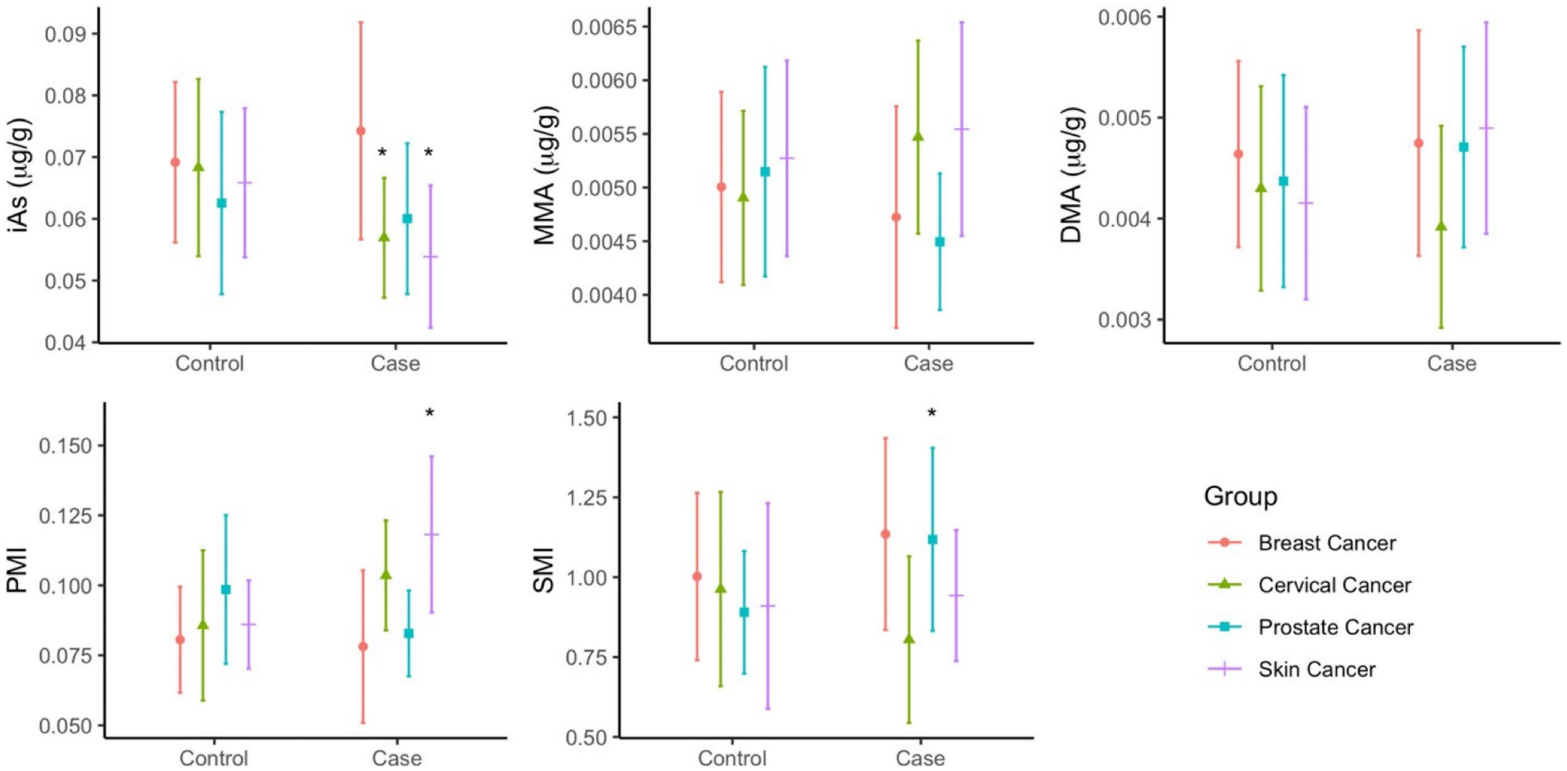
Mean concentrations (µg/g) of As species (iAs, MMA, DMA) and As methylation index (PMI, SMI) in toenails of cancer cases and controls. Statistical significance (p<0.05) is indicated by *.

The predominant form of arsenic found in toenails was iAs, accounting for more than 80% of total arsenic. Moreover, when compared to their respective controls, the concentrations of iAs were significantly higher among both cervical and skin cancer cases (Figure 2). Mean concentrations of arsenic species (Supplementary Table S2) were more varied between cancer cases than between controls: iAs ranged from 0.054–0.074 μg/g, MMA ranged from 0.0045–0.0055 μg/g, and DMA ranged from 0.0039–0.0049 μg/g. While the concentrations of MMA and DMA in toenails did not significantly differ between cases and controls in any of the groups, the PMI (ratio of MMA to iAs) and SMI (ratio of DMA to MMA) was significantly different between cases and controls in the skin and cervical cancer groups, respectively.

**Figure 3:**
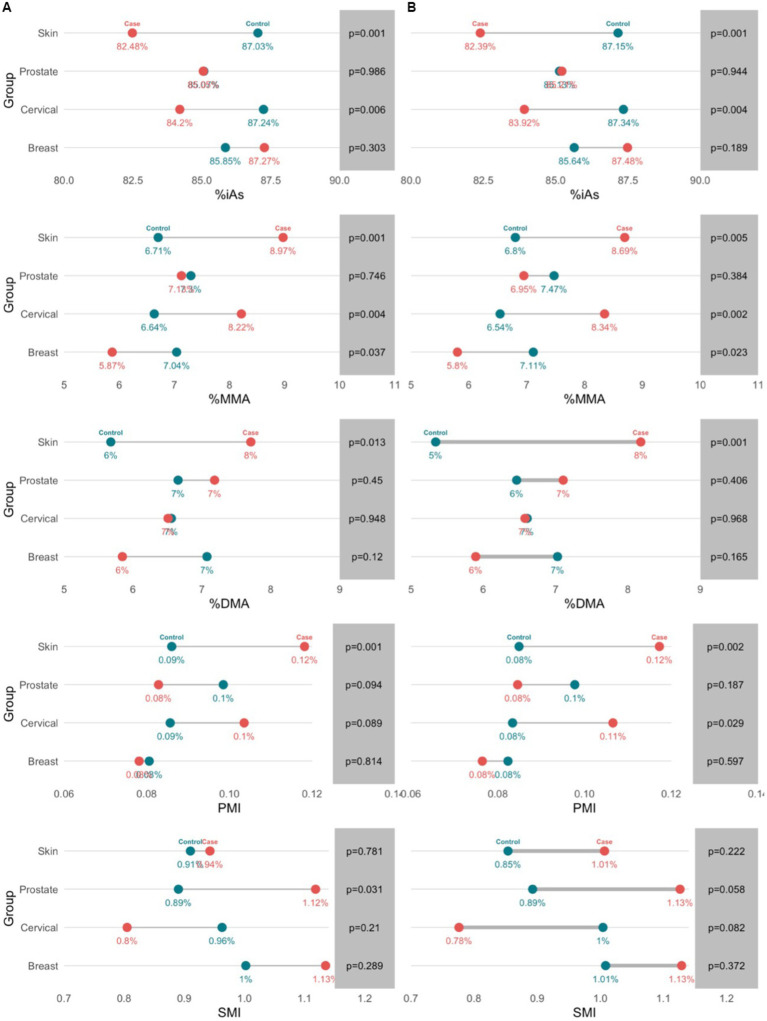
Profiles of toenail arsenic species between cancer cases and controls in unadjusted (a) and adjusted (b) models. Arsenic speciation profiles were adjusted for age, sex, family history of cancer, province of residence, water source, physical activity, and smoking.

In multivariate analysis, arsenic speciation profiles were significantly different between cases and controls in the breast cancer (*p* = 0.0330), cervical cancer (*p* = 0.0228), and skin cancer (*p* = 0.0085) groups (Supplementary Table S3). The arsenic speciation profiles patterns were not uniform across groups, and significant mean differences were observed among different variables across the groups. Adjustment did not appreciably alter the observed mean differences, but many increased in magnitude (Figure 3). The adjusted models for the breast, cervical, and skin cancer groups indicated the selected covariates were not significant predictors of arsenic speciation. By contrast, the adjusted model for the prostate cancer group was significant (*p* = 0.0373); significant predictors of arsenic speciation were province of residence (*p* = 0.0004) and smoking status (*p* = 0.0095). However, after adjustment, none of the arsenic speciation variables were statistically significantly different between prostate cancer cases and controls.

### 3.2. Metallomes profiles

**Figure 4:**
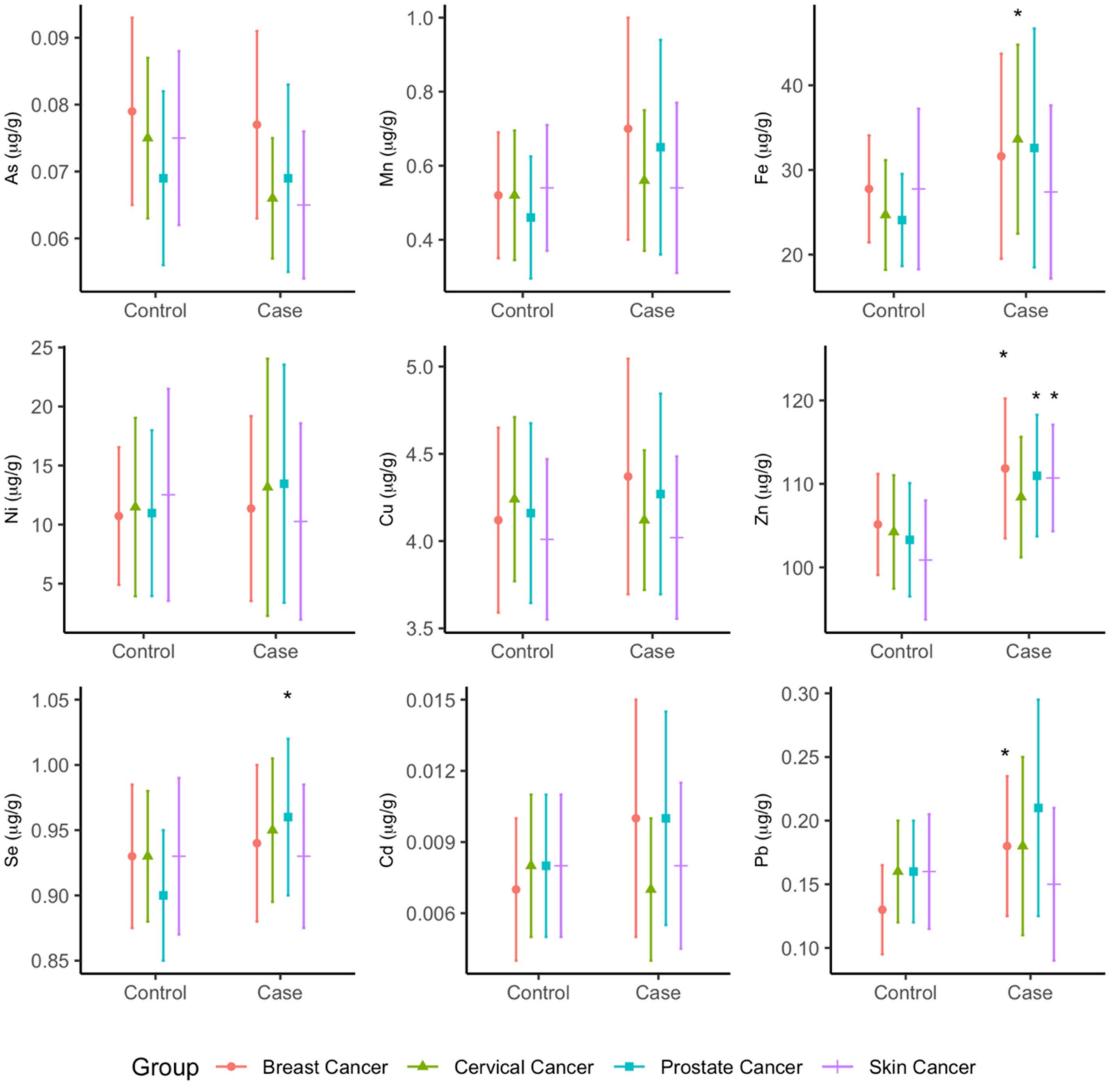
Mean total concentrations (µg/g) of metallome in toenails of cancer cases and controls. Statistical significance (p<0.05) is indicated by *.

Significantly higher concentrations of zinc were measured among cases compared to controls in the breast (*p* = 0.0412), prostate (*p* = 0.0116), and skin (*p* = 0.0011) cancer groups (Figure 4). In the breast cancer group, lead concentrations were higher among cases compared to controls (*p* = 0.0385). In the cervical cancer group, higher concentrations of iron were observed among cases compared to controls (*p* = 0.0291). Prostate cancer cases had statistically significantly higher selenium concentrations (*p* = 0.0116) when compared to their controls (Figure 4). The means and standard deviations (SD) of the arsenic species (μg/g), methylation indices (PMI, SMI), and total metallomes (μg/g) are provided in Supplementary Information (Supplementary Table S1).

In multivariate analysis, profiles of metallomes were significantly different between cases and controls in the prostate cancer (*p* = 0.0244) and skin cancer (*p* = 0.0321) groups in unadjusted analyses, whereas the breast and cervical cancer models were non-significant. However, significantly higher mean concentrations of zinc were observed in the breast cancer (*p* = 0.041), prostate cancer (*p* = 0.012), and skin cancer (*p* = 0.001) cases, higher mean iron was observed in cervical cancer cases (*p* = 0.029), and higher mean selenium was observed in prostate cancer cases (*p* = 0.012). The mean differences did not appreciably change between unadjusted and adjusted analysis (Supplementary Table S4). In the MANCOVA analysis, only the prostate cancer model remained statistically significant (*p* = 0.0194), and significant differences in zinc and selenium concentrations between cases and controls persisted. None of the covariates in this model were significant predictors of metallome profiles.

### 3.3. Metallomes correlation

**Figure 5:**
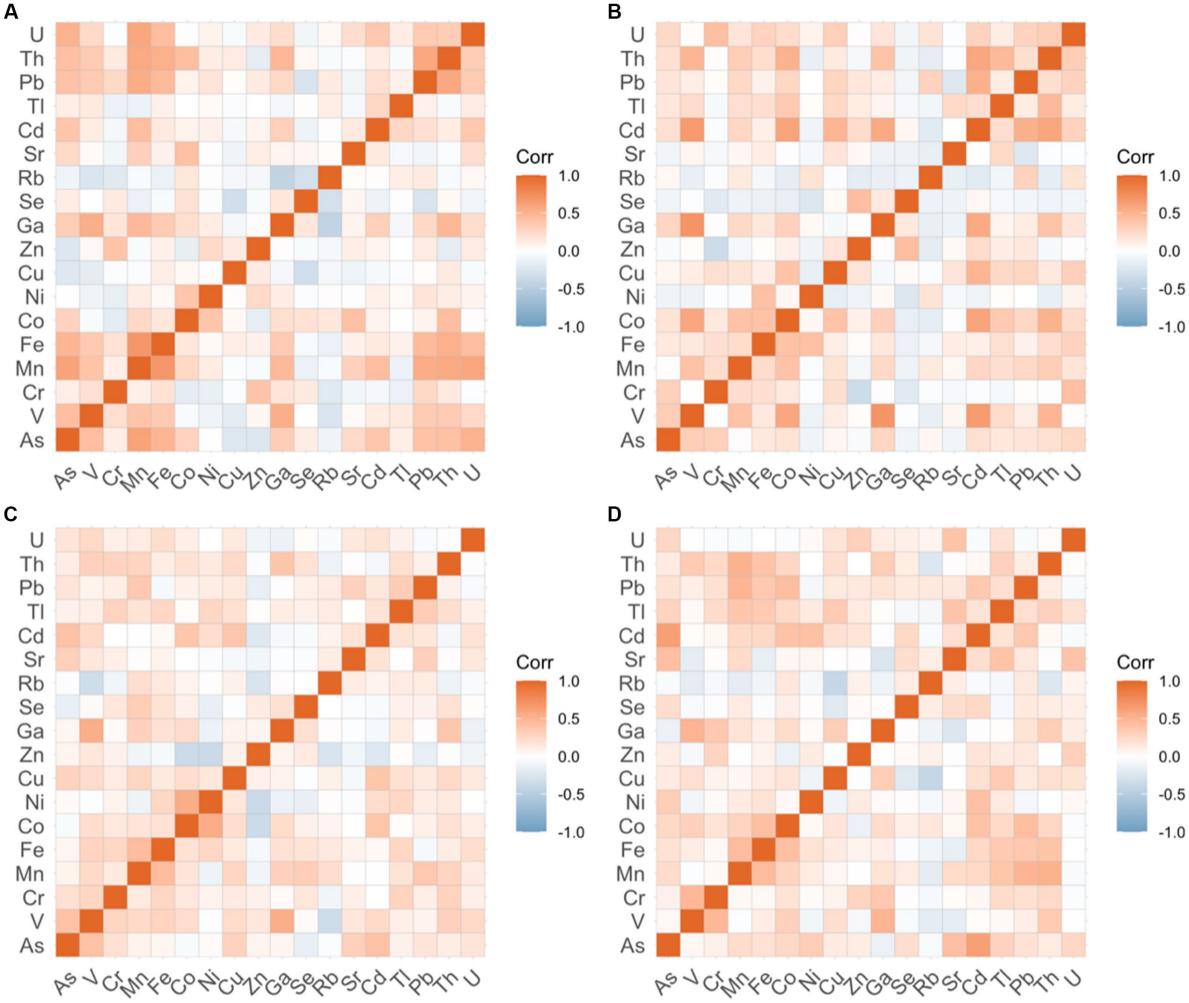
Correlation of toenail metallomic profiles in cancer cases.

Of the 23 elements measured, only 18 were consistently above the MDLs (V, Cr, Mn, Fe, Co, Ni, Cu, Zn, Ga, As, Se, Rb, Sr., Cd, Tl, Pb, Th, and U). The concentrations of several metals (metallomes) were significantly correlated in many of the groups (Figure 5). Among breast cancer cases, significant positive correlations were observed in the concentration of arsenic-manganese (*r* = 0.61, *p* = 0.001) and manganese-lead (*r* = 0.55, *p* = 0.0081), whereas positive correlations between cadmium-lead (*r* = 0.52, *p* = 0.0157) and arsenic-cadmium (*r* = 0.60, *p* = 0.0010) were observed among the cervical cancer cases. Significant correlations were also observed between copper-lead in the breast cancer control group (*r* = 0.54, *p* = 0.0082) and in cervical cancer control groups (*r* = 0.49, *p* = 0.0379). In addition, the concentrations of manganese-iron were positively and significantly correlated in both the cervical cancer control group (*r* = 0.59, *p* = 0.0015) and the prostate cancer control group (*r* = 0.47, *p* = 0.0465), while manganese-lead were correlated in both the cervical cancer (*r* = 0.54, *p* = 0.0091) and skin cancer (*r* = 0.50, *p* = 0.0250) control groups. Significant positive correlations were also observed in the concentrations of iron-copper (*r* = 0.49, *p* = 0.0295) and iron-lead (*r* = 0.54, *p* = 0.0072) in the skin cancer control group.

## 4. Discussion

### 4.1. Arsenic speciation profiles are associated with breast, cervical, and skin cancers

The proportions of arsenic species measured in toenail samples are markedly different than those previously measured in urine, but are comparable to those found in toenails (33, 49, 50). Our previous work has found that toenails better capture arsenic speciation profiles and exposure to iAs when compared to urine samples (33). The compositional differences of arsenic species in these biomarkers may be explained by differential exposure levels or abilities to process iAs between study populations, or differences in the properties of the metabolites themselves. For example, iAs (III) has been shown to have a strong affinity to keratin (51). It is likely these biomarkers are complementary measures of arsenic exposure, but given our previous findings, toenails appear to be a more advantageous biomarker for evaluating arsenic methylation capacity, and thus, the risk of associated cancers.

#### 4.1.1. Profiles of arsenic speciation by cancer group

There was a statistically significant difference in the arsenic speciation profiles of those with a history of breast cancer and those without (*p* = 0.033). In particular, those with breast cancer history had a statistically significantly lower %MMA compared to controls (*p* = 0.037). This is one of the first studies to assess arsenic speciation among those with a history of breast cancer. However, some previous research has found that increased urinary %MMA was significantly associated with an increased risk of breast cancer (52, 53). These results provide further evidence that arsenic speciation profiles differ between urine and toenails. In addition, the profile pattern of %MMA in the breast cancer group differed from the profile pattern observed in the other groups, suggesting that speciation and methylation also differ by cancer type. Experimental evidence has generated several proposed mechanisms of effect of arsenic-induced breast cancer, which include iAs (III) induced ROS generation, DNA oxidative damage, metallothioneine and c-Myc proteins, NF-kB activation, and cell proliferation in human breast cancer MCF-7 cells, among others (54). There is also evidence of genetic polymorphisms affecting both methylation capacity and breast cancer risk. In particular, some polymorphisms may provide protective benefits against arsenic related breast cancers (55).

Cervical cancer cases had significantly lower %iAs compared to controls (*p =* 0.0058), as well as significantly higher %MMA compared to controls (*p =* 0.0042). To the best of our knowledge, this is the first study to measure arsenic speciation among those with a history of cervical cancer. The human papillomavirus is considered the primary cause of cervical cancer (56); however, there is preliminary evidence to suggest that oxidative stress may play a role (57). Arsenic is known to induce oxidative stress through the production of ROS (8, 54). While not the primary cause, arsenic exposure and methylation capacity may contribute to the development of cervical cancer. Further research is necessary to better understand this association, and to confirm the results found in the present study.

The results of our study show that in the prostate cancer group, the SMI was statistically significantly higher among cases compared to controls (*p* = 0.0307). These findings are consistent with previous results from our lab comparing arsenic speciation and metallome profiles in prostate cancer cases and matched controls using both toenail and urine samples (33). Specifically, in our previous work, we found that %MMA and PMI were lower among prostate cancer cases compared to controls, and the SMI was higher. Both present and previous work employed a similar study design, although the sample size of our previous work was much larger. Given that the target population is the same, the similarity in findings was expected. However, the smaller sample size in the present study may explain the lack of association in the present study for %MMA and PMI. This highlights the need for larger sample sizes in all groups in future work.

Our results also showed that in the skin cancer group, cases had significantly lower %iAs (*p* = 0.0006), and significantly higher %MMA (*p* = 0.0008) and %DMA (*p* = 0.0127) compared to controls. The PMI was also significantly higher among skin cancer cases compared to controls (*p* = 0.0015). These results are somewhat consistent with the existing, albeit scarce literature pertaining to arsenic speciation profiling in toenails. Our group previously found similar, albeit not statistically significant arsenic species profile patterns for skin cancer cases, however this may be attributed to the small samples sizes of that study (34). Other, independent works on skin cancer and arsenic speciation has generally supported our findings: that a higher proportion of the MMA species is associated with skin cancer. For example, one study found that when compared to controls, skin cancer cases had higher urinary %MMA, as well as higher urinary %iAs and lower urinary %DMA (58). However, in another study, no significant associations between %iAs, %MMA, %DMA, PMI and SMI and skin cancer were found. Instead, given a low SMI (ratio of DMA:MMA), a higher cumulative arsenic exposure was associated with increased risk of skin cancer (59).

While the proportions of arsenic species (particularly %iAs and %DMA) differ between toenail and urine biomarkers, the results of this study and previous research indicates that higher %MMA is linked to increased skin cancer risk (58). Higher stored or excreted %MMA may be indicative of a lower methylation capacity or ability, as the methylation process is incomplete, which has been associated with higher risk of arsenic-induced skin malignancies (60). Despite some inconsistencies in the literature, the evidence linking arsenic speciation and skin cancer is quite strong. Based on the findings of this study, we conclude that arsenic speciation among those with skin cancer is different from those without. In addition, these findings indicate that toenail biomarkers are a viable option for monitoring arsenic speciation. In the future, with additional evidence, toenail biomarkers could potentially be used as a prognostic tool.

#### 4.1.2. External factors not significant predictors of arsenic speciation profiles

Of the cancer groups analyzed, the adjusted models were statistically significant for only the skin cancer group (*p* = 0.0458). However, despite the model being significant, none of the covariates included were statistically significant predictors in the present study. This provides further evidence of the association between methylation capacity and cancer and supports the use of toenail arsenic species as potential biomarkers. However, the lack of association between the covariates included in the adjusted model (family history of cancer, province of residence, water source, physical activity, smoking, and BMI) indicated that these factors did not affect arsenic methylation capacity in this population group. This contrasts with evidence that biological, lifestyle, and environmental factors are linked with arsenic methylation capacity, but it is limited and often inconsistent. For example, those with higher BMI may methylate arsenic more efficiently and have a lower arsenic body burden (50, 61), while smoking may be associated with decreased arsenic methylation capacity (62). Other factors that have been related to arsenic methylation in humans include ethnicity, arsenic exposure dosage, age, sex, pregnancy, and breastfeeding (61). While the present study found no significant associations between the selected covariates and arsenic speciation, this may be attributed to the study population—clearer associations may be revealed in a more diverse study population. The present study found weak evidence linking some of these factors to arsenic speciation profiles, and as such, cannot make conclusive statements regarding these associations without further investigation.

### 4.2. Metallome profiles analysis revealed higher zinc concentrations in cancer cases

Results here show significantly higher toenail zinc concentrations among breast, prostate, and skin cancer cases compared to controls. The current evidence linking zinc and cancer in general highlights the adverse effect of deficiencies (26). One study (63) measuring trace metals in fingernails of breast cancer cases and controls found similar zinc concentrations in their control group (103.24, SD = 38.96 μg/g versus 105.15, SD = 12.10 μg/g in the present study), but much lower concentrations among breast cancer patients (70.06, SD = 24.12 μg/g versus 111.85, SD = 16.76 μg/g in the present study). Another study found comparable zinc nail concentrations in prostate cancer patients (114.2, standard error = 4.53 μg/g) but non-significant differences between cases and controls (64). The differences between cases and controls we observed here may be explained by the varied zinc levels across populations, as well as differences in case definition (29, 65). For example, using a metallomics approach, Schilling and colleagues (2020) explained that higher urinary zinc concentrations may be caused by the downregulation of metal-binding transporters that promote the development and growth of cancer cells. In the results we report here, higher nail-zinc concentrations could be indicative of poor uptake of zinc, and homeostasis may be important in pathogenesis (24). However, critical gaps remain on zinc toxicokinetics and the associations between zinc levels and its association with cancer. Previous research has indicated that the degree of zinc exposure may play a key role in determining its association with cancer. Specifically, the effect of zinc deficiency has been shown to be different from overexposure, but both may increase cancer risk. While a deficiency in zinc may be a risk factor due to its antioxidant properties, over-exposure to zinc has been linked with skin cancers (66).

The findings of this research showed higher toenail-lead concentrations among breast cancer cases compared to controls. Lead concentrations were higher among cervical and prostate cancer cases compared to their respective controls, but the difference was not statistically significant. Another study found prostate cancer cases had statistically non-significant higher lead concentrations in nails compared to controls, but the concentrations were much higher (100-fold) in both groups compared to the present study (64).

Despite limited evidence from human and animal studies, the IARC has classified lead as a possible carcinogen (1, 67), and there is some evidence to suggest there may be an association with lung, stomach, and brain cancers (32). In addition, little research has been conducted on the use of nails as a biomarker for lead exposure. Our findings suggest there may be an association between lead concentrations and breast cancer. This result is consistent with a previous study of metallome in breast cancer patients (25). However, further research is required to ascertain the ability of toenails to both measure lead body burden, and to assess potential interactions between lead and other metals, such as arsenic.

The present study found no association between toenail cadmium concentration and cancer, despite there being some limited evidence to suggest cadmium is associated with lung, prostate, bladder, and breast cancers (68, 69). Some observational studies using nail samples have found cadmium concentrations to be higher in prostate cancer cases than in healthy controls (64, 70). Nail samples are considered a reliable biomarker for cadmium exposure, especially as they capture cadmium accumulation over a long period of time (71, 72). However, in the present study, toenail cadmium concentrations (0.007–0.010 μg/g) were much lower compared to those (1.11 ± 0.83 μg/g) previously found in nail samples (68). Considered together, the lack of observed association between cadmium and the cancer types in this study may be the result of low cadmium exposure among Atlantic Canadian. However, further research is needed.

Results we obtained show no association between toenail total arsenic concentrations and cancer. Arsenic exposure has been previously associated with liver, lung, urinary bladder, skin, kidney, breast, and prostate cancers using other biomarkers (8, 73). Recent evidence, however, has found that arsenic metabolism is the key mechanism behind arsenic-related cancers *via* the genetic polymorphisms involved in the process and the varied toxicity of the metabolites (74, 75). Our findings that arsenic speciation profiles—not total arsenic concentrations—are linked to history of several cancer types, provide further evidence for the important role of arsenic methylation and the need to use relevant, long-term biomarkers that can capture the variation in these profiles.

#### 4.2.1. Presence of known toxic metals—arsenic, cadmium, lead, manganese—are correlated

The total concentrations of several metals (arsenic, manganese, iron, lead, cadmium, copper) were significantly correlated, but patterns varied by group. These metals represent common water contaminants that are often found together in groundwater systems (76). Most existing metal analyses have focused on the excess or deficiency of specific metals, while little research on the interrelationship between concentrations has been examined. This is a critical gap in our understanding, as we know that metals can have antagonistic or synergistic effects (77). For example, selenium has been shown to be positively associated with arsenic methylation capacity (78). Nevertheless, previous work have found statistically significant correlations between similar metals (cadmium, copper, lead, zinc, manganese) measured in nail samples were observed in a study of oral cancer patients (79). Similarly, Burton and colleagues (25) observed correlations between cadmium with arsenic, zinc, copper, and other metals, and between lead and selenium, nickel, and zinc. Further investigation is required to elucidate the interrelationship between essential and non-essential metals and their role in cancer development. For this reason, the development of a metallomic profile chart that indicates target ranges is critical as it could be key in preventing and biomonitoring populations for cancer, among other heavy-metal-related chronic diseases.

### 4.3. Strengths and limitations

The strengths of this study include the use of a novel toenail biomarker, new analytical methods for toenail arsenic species and metallomes, and the large Atlantic PATH cohort to assess arsenic and trace metal exposure and their roles in less studied arsenic-related cancers. Currently, urine is the gold-standard biomarker to measure total concentrations of trace metals, however the use of urine is limited as it only captures a short exposure window. For the purposes of monitoring chronic exposure, as is the case for cancer risk factors, this is a critical shortcoming. The advantages of using toenails include non-invasive sample collection, and the ease and duration of sample storage. In addition, this research has used a newly developed analytical methods to measure arsenic species in toenails, which yields higher extraction efficiencies than previously observed. Moreover, this approach undergone method validation using two certified reference materials. To our knowledge, this study fills a major gap in the literature and is the first to investigate arsenic exposure and arsenic speciation among cervical cancer cases, and among the first to do so with toenails among breast, prostate, and skin cancers. Furthermore, this research is some of the first to employ a metallomics approach using toenails to compare profiles between cases and controls in several cancer groups.

Nonetheless, we note several limitations. The data in this study are cross-sectional and rely on prevalent cancer cases, and without temporal order, there is no way to ascertain causality between arsenic exposure/speciation and cancer. This limitation will be addressed in subsequent studies thanks to the longitudinal nature of Atlantic PATH, which will allow the use of incident cases. In addition, the data do not distinguish between melanoma and non-melanoma skin cancers. Many of the covariates used in this study were sourced from the questionnaire and some had missing data. Given the nature of the Atlantic PATH cohort, these findings may not be generalizable to the entire general population. The observed differences in the Atlantic PATH participants compared to the general population include higher overall socioeconomic status and higher representations of women and people with university degrees. These differences are important as those with higher incomes and levels of scientific literacy may be more likely to engage in remediation strategies for well water as a primary source of drinking water. Finally, we believe that drinking water is the primary source of exposure to arsenic, but do not have information on the duration of the exposure.

### 4.4. Conclusion

Despite these limitations, this study makes important contributions to the current literature. Exposure to arsenic and other heavy metals through contaminated drinking water remains a problem experienced in Atlantic Canada and around the world. While the mechanisms of arsenic toxicity and carcinogenicity are yet to be fully elucidated, this research provides further evidence of the potential mediating role that arsenic species may play in cancer development. The findings of our work provide further evidence that arsenic species, rather than total arsenic, is critical in understanding cancer risk associated with arsenic exposure. In particular, our findings support the hypothesis that individuals with cancer have differential arsenic speciation and metallomics profiles compared to healthy controls, indicating that environmental arsenic exposure plays a role in carcinogenesis. In addition, the research we present here suggests that arsenic speciation profiles may differ slightly by cancer type. Further research is required to confirm these findings, to better understand the mechanisms underlying this variation, and to develop upstream population health monitoring tools in response to environmental arsenic and metallome exposure.

Future research should attempt to gain a better understanding on the association between arsenic and metal exposures and cancer. Specifically, more exposure biomarker studies using a biological sample for chronic exposure are needed to understand how arsenic exposure induces cancer. Furthermore, additional method development for arsenic speciation analysis in toenails should focus on the ability to differentiate between trivalent and pentavalent arsenicals in biomarkers that would provide a more comprehensive understanding of methylation and its role in toxicity. From a population health and epidemiological perspective, the next steps include incident case analysis, accounting for the time elapsed since the first cancer diagnosis, and a larger sample. Our future research will include the use of incident cases and larger sample numbers which help to determine more definitive inferences about potentially causal mechanisms. Furthermore, our findings were consistent with previous evidence that metallome concentrations are interrelated and may generate synergistic or protective effects. As such, future research should analyze arsenic speciation and important metallomes together in profile analysis to determine whether there are any associations or interactions with methylation capacity. Lastly, given some higher arsenic and metallome profiles found in this study among cancer survivors compared to controls and their link with oxidative stress, future studies should attempt to examine their role as a possible environmental mediator of poor oncological outcomes following active forms of cancer treatments.

## Data availability statement

The original contributions presented in the study are included in the article/Supplementary material, further inquiries can be directed to the corresponding author.

## Ethics statement

The studies involving human participants were reviewed and approved by Dalhousie Research Ethics Board (#2016–3,896). The patients/participants provided their written informed consent to participate in this study.

## Author contributions

The conception of this work was led by JK, with contribution from KH, ES, and GI. JK, KH, ES, and GI designed the study. KH analyzed the toenail samples with assistance from EK. KH performed all statistical and data analysis, interpreted the data, drafted the manuscript and revised it. All authors contributed to the article and approved the submitted version.

## Funding

This research was supported by Nova Scotia Health Authority (NSHA) and Beatrice Hunter Cancer Research Institute (BHCRI). The data used in this research were made available by the Atlantic Partnership for Tomorrow’s Health (Atlantic PATH) study, which is the Atlantic Canada regional component of the Canadian Partnership for Tomorrow’s Health funded by the Canadian Partnership Against Cancer and Health Canada. The views expressed herein represent the views of the authors and do not necessarily represent the views of Health Canada.

## Conflict of interest

The authors declare that the research was conducted in the absence of any commercial or financial relationships that could be construed as a potential conflict of interest.

## Publisher’s note

All claims expressed in this article are solely those of the authors and do not necessarily represent those of their affiliated organizations, or those of the publisher, the editors and the reviewers. Any product that may be evaluated in this article, or claim that may be made by its manufacturer, is not guaranteed or endorsed by the publisher.
