# Supplementary material for: Toenail arsenic species and metallome profiles associated with breast, cervical, prostate, and skin cancer prevalence in the Atlantic Partnership for Tomorrow’s Health cohort
Source: Front Public Health. 2023 Jun 15;11:1148283. doi: 10.3389/fpubh.2023.1148283 (PMC10308375; doi:10.3389/fpubh.2023.1148283)
Supplement: Supplementary file 1 [file Data_Sheet_1.docx]

**Supplementary Information**

Table S1 Method detection limits for metals and arsenic species.

| **Analyte** | **MDL (μg/L)** | **MDL (μg)^a^** | **MDL (μg/g)^b^** |
| --- | --- | --- | --- |
| V | 0.009 | 0.00009 | 0.003 |
| Cr | 0.33 | 0.0033 | 0.10 |
| Mn | 0.081 | 0.0008 | 0.025 |
| Fe | 5.8 | 0.058 | 1.83 |
| Co | 0.027 | 0.0003 | 0.009 |
| Ni | 0.35 | 0.0035 | 0.11 |
| Cu | 0.79 | 0.0079 | 0.25 |
| Zn | 3.7 | 0.037 | 1.16 |
| Ga | 0.0047 | 0.00005 | 0.0015 |
| As | 0.011 | 0.00011 | 0.0035 |
| Se | 0.019 | 0.0002 | 0.0060 |
| Rb | 0.033 | 0.00033 | 0.010 |
| Sr | 0.10 | 0.0010 | 0.032 |
| Cd | 0.016 | 0.00016 | 0.0051 |
| Tl | 0.0003 | 0.000003 | 0.0001 |
| Pb | 0.15 | 0.0015 | 0.047 |
| Th | 0.0007 | 0.000007 | 0.00022 |
| U | 0.0007 | 0.000007 | 0.00022 |
| MMA | 0.0049 | 0.000049 | 0.0015 |
| DMA | 0.0045 | 0.000045 | 0.0014 |
| iAs | 0.017 | 0.00017 | 0.0052 |

*Notes:* Metal and iAs MDLs were calculated according to EPA procedure using method blanks. MMA and DMA MDLs were calculated using 7 replicates of the lowest level calibration standard (0.02 μg/L). ^a^ Calculated by multiplying the MDL in μg/L by the total sample volume (0.010 L for total metal analysis; 0.01005 L for arsenic speciation analysis). ^b^ Calculated by multiplying the MDL in μg by average sample mass (0.0391 g).

Table S2 Means and standard deviations of arsenic species (%), methylation indices, and total metallome concentrations (ug/g) by case/control group.

|  | **Breast** **Cancer**  **Group (n=82)**  Mean *(SD)* | | **Cervical Cancer Group (n=82)**  Mean *(SD)* | | **Prostate Cancer Group (n=88)**  Mean *(SD)* | | **Skin Cancer Group (n=86)**  Mean *(SD)* | |
| --- | --- | --- | --- | --- | --- | --- | --- | --- |
|  | Case | Control | Case | Control | Case | Control | Case | Control |
| **As speciation** | | | | | | | | |
| %iAs | 87.06 *(6.03)* | 86.81 *(4.76)* | 85.11 *(4.22)* | 86.73 *(6.05)* | 85.72 *(4.38)* | 85.35 *(6.28)* | **82.37 *(6.39)*** | **86.65 *(4.08)*** |
| %MMA | 6.33 *(3.07)* | 6.83 *(2.72)* | **8.71 *(2.78)*** | **7.01 *(3.19)*** | 6.95 *(2.13)* | 8.05 *(3.75)* | **9.44 *(3.68)*** | **7.30 *(2.12)*** |
| %DMA | 6.61 *(3.66)* | 6.35 *(2.89)* | 6.18 *(2.80)* | 6.26 *(3.61)* | 7.33 *(3.22)* | 6.60 *(3.21)* | **8.19 *(3.81)*** | **6.05 *(3.27)*** |
| PMI | 0.07 *(0.04)* | 0.08 *(0.04)* | **0.10 *(0.04)*** | **0.08 *(0.04)*** | 0.08 *(0.03)* | 0.10 *(0.05)* | **0.12 *(0.05)*** | **0.08 *(0.03)*** |
| SMI | 1.10 *(0.46)* | 0.98 *(0.43)* | 0.78 *(0.48)* | 0.95 *(0.53)* | **1.11 *(0.53)*** | **0.88 (*0.37)*** | 0.94 *(0.45)* | 0.88 *(0.50)* |
| **Total Metals (ug/g)** | | | | | | | | |
| As | 0.077 *(0.028)* | 0.079 *(0.028)* | 0.066 *(0.018)* | 0.075 *(0.024)* | 0.069 *(0.028)* | 0.069 *(0.026)* | 0.065 *(0.022)* | 0.075 *(0.026)* |
| Mn | 0.70 *(0.60)* | 0.52 *(0.34)* | 0.56 *(0.38)* | 0.52 *(0.35)* | 0.65 *(0.58)* | 0.46 *(0.33)* | 0.54 *(0.46)* | 0.54 *(0.34)* |
| Fe | 31.62 *(24.24)* | 27.76 (*12.66)* | **33.64 *(22.34)*** | **24.68 (*12.97)*** | 32.60 *(28.24)* | 24.08 (*10.90)* | 27.40 *(20.48)* | 27.75 (*18.99)* |
| Ni | 11.36 *(15.65)* | 10.72 *(11.67)* | 13.16 *(21.78)* | 11.48 *(15.11)* | 13.46 *(20.17)* | 10.97 *(14.05)* | 10.26 *(16.62)* | 12.52 *(17.95)* |
| Cu | 4.37 *(1.35)* | 4.12 *(1.06)* | 4.12 *(0.80)* | 4.24 *(0.94)* | 4.27 *(1.15)* | 4.16 *(1.03)* | 4.02 *(0.93)* | 4.01 *(0.92)* |
| Zn | **111.85 *(16.76)*** | **105.15 *(12.10)*** | 108.42 *(14.43)* | 104.24 *(13.57)* | **110.99 *(14.56)*** | **103.30 *(13.57)*** | **110.71 *(12.76)*** | **100.88 *(14.27)*** |
| Se | 0.94 (*0.12)* | 0.93 (*0.11)* | 0.95 (*0.11)* | 0.93 (*0.10)* | **0.96 (*0.12)*** | **0.90 (*0.10)*** | 0.93 (*0.11)* | 0.93 (*0.12)* |
| Cd | 0.010 *(0.010)* | 0.007 *(0.006)* | 0.007 (0.006*)* | 0.008 (0.006*)* | 0.010 (0.009*)* | 0.008 (0.006*)* | 0.008 *(0.007)* | 0.008 *(0.006)* |
| Pb | **0.18 *(0.11)*** | **0.13 (*0.07)*** | 0.18 *(0.14)* | 0.16 (*0.08)* | 0.21 *(0.17)* | 0.16 (*0.08)* | 0.15 *(0.12)* | 0.16 (*0.09)* |

Notes: **bolded** values indicate significant difference between cases and controls (*p* < 0.05).

Table S3 MANOVA analyses comparing As speciation profiles between cases and control, by group, sampled from the Atlantic PATH cohort study (2009-2015), N=392.

|  | **Mean difference *(SE)*** | **p-value** | **Mean difference *(SE)*** | **p-value** | **Effect size^a^** |
| --- | --- | --- | --- | --- | --- |
| **Breast Cancer (n=82)** | | | | | |
|  | Unadjusted Model (**p = 0.033)** | | Adjusted Model^b^ (p = 0.1727) | |  |
| %iAs | 1.41 *(1.36)* | 0.303 | 1.84 *(1.39)* | 0.189 | 0.03 |
| %MMA | -1.17 *(0.55)* | **0.037** | -1.31 *(0.56)* | **0.023** | 0.08 |
| %DMA | -1.23 *(0.78)* | 0.120 | -1.13 *(0.81)* | 0.165 | 0.03 |
| PMI | -0.002 *(0.010)* | 0.814 | -0.01 *(0.01)* | 0.597 | >0.01 |
| SMI | 0.13 *(0.12)* | 0.289 | 0.12 *(0.13)* | 0.372 | 0.01 |
| **Cervical Cancer (n=82)** | | | | | |
|  | Unadjusted Model (p = 0.0228) | | Adjusted Model^b^ (p = 0.1976) | |  |
| %iAs | -3.04 *(1.07)* | **0.006** | -3.43 *(1.16)* | 0.004 | 0.12 |
| %MMA | 1.58 *(0.54)* | **0.004** | 1.80 *(0.56)* | 0.002 | 0.14 |
| %DMA | -0.05 *(0.71)* | 0.948 | -0.03 *(0.73)* | 0.968 | <0.01 |
| PMI | 0.02 *(0.01)* | 0.089 | 0.02 *(0.01)* | 0.029 | 0.07 |
| SMI | -0.16 *(0.12)* | 0.210 | -0.23 *(0.13)* | 0.082 | 0.05 |
| **Prostate cancer (n=88)** | | | | | |
|  | Unadjusted Model (p = 0.2750) | | Adjusted Model^b^ (**p = 0.0373**) | |  |
| %iAs | -0.02 *(1.02)* | 0.986 | 0.08 *(1.17)* | 0.944 | <0.001 |
| %MMA | -0.17 *(0.52)* | 0.746 | -0.52 *(0.59)* | 0.384 | 0.01 |
| %DMA | 0.53 *(0.70)* | 0.450 | 0.64 *(0.77)* | 0.406 | 0.01 |
| PMI | -0.02 *(0.01)* | 0.094 | -0.01 *(0.01)* | 0.187 | 0.03 |
| SMI | 0.23 *(0.10)* | **0.031** | 0.23 *(0.12)* | 0.058 | 0.05 |
| **Skin Cancer (n=86)** | | | | | |
|  | Unadjusted Model (**p = 0.0085**) | | Adjusted Model^b^ (p = 0.0799) | |  |
| %iAs | -4.55 *(1.27)* | **0.001** | -4.76 *(1.39)* | **0.001** | 0.15 |
| %MMA | 2.27 *(0.65)* | **0.001** | 1.89 *(0.65)* | **0.005** | 0.11 |
| %DMA | 2.03 *(0.80)* | **0.013** | 2.84 *(0.85)* | **0.001** | 0.14 |
| PMI | 0.03 *(0.01)* | **0.001** | 0.03 *(0.01)* | **0.002** | 0.14 |
| SMI | 0.03 *(0.12)* | 0.781 | 0.15 *(0.12)* | 0.222 | 0.02 |

^a^ Partial $\eta^{2}$ as a measure of effect size. ^b^ Model adjusted for age, family history of cancer, province of residence, water source, physical activity, smoking status, and BMI.

Table S4 MANOVA analyses comparing profiles of metallomes between cases and control, by group, sampled from the Atlantic PATH cohort study (2009-2015), N=392.

|  | **Mean difference *(SE)*** | **p-value** | **Effect Size** | **Mean difference *(SE)*** | **p-value** | **Effect size^a^** |
| --- | --- | --- | --- | --- | --- | --- |
| **Breast Cancer (n=82)** | | | | | | |
|  | Unadjusted Model (***p* = 0.3085**) | | | Adjusted Model^b^ (*p* = 0.5409) | | |
| As | -0.002 (0.006) | 0.704 | 0.002 | -0.001 (0.006) | 0.836 | 0.001 |
| Mn | 0.18 (0.11) | 0.093 | 0.04 | 0.18 (0.11) | 0.104 | 0.04 |
| Fe | 3.87 (4.27) | 0.368 | 0.01 | 4.10 (4.41) | 0.356 | 0.01 |
| Ni | 0.64 (3.05) | 0.835 | <0.001 | 1.27 (2.98) | 0.670 | 0.003 |
| Cu | 0.25 (0.27) | 0.359 | 0.01 | 0.24 (0.29) | 0.409 | 0.01 |
| Zn | 6.70 (3.23) | **0.041** | 0.05 | 6.89 (3.52) | 0.055 | 0.06 |
| Se | 0.003 (0.025) | 0.911 | <0.001 | -0.001 (0.03) | 0.982 | <0.001 |
| Cd | 0.003 (0.002) | 0.123 | 0.03 | 0.003 (0.002) | 0.176 | 0.03 |
| Pb | 0.04 (0.02) | **0.038** | 0.05 | 0.04 (0.02) | 0.058 | 0.05 |
| **Cervical Cancer (n=82)** | | | | | | |
|  | Unadjusted Model (*p* = 0.1198) | | | Adjusted Model^b^ (*p* = 0.3699) | | |
| As | -0.01 (0.005) | 0.081 | 0.04 | -0.01 (0.005) | 0.169 | 0.03 |
| Mn | 0.05 (0.08) | 0.551 | 0.004 | 0.09 (0.08) | 0.315 | 0.02 |
| Fe | 8.96 (4.03) | 0.029 | 0.06 | 7.99 (4.25) | 0.065 | 0.05 |
| Ni | 1.68 (4.14) | 0.686 | 0.002 | 1.63 (4.66) | 0.727 | 0.002 |
| Cu | -0.13 (0.19) | 0.518 | 0.005 | -0.24 (0.20) | 0.236 | 0.02 |
| Zn | 4.18 (3.09) | 0.180 | 0.02 | 3.33 (3.26) | 0.311 | 0.02 |
| Se | 0.03 (0.02) | 0.247 | 0.02 | 0.03 (0.02) | 0.215 | 0.02 |
| Cd | -0.001 (0.001) | 0.698 | 0.002 | -0.0004 (0.001) | 0.793 | 0.001 |
| Pb | 0.02 (0.02) | 0.380 | 0.01 | 0.02 (0.03) | 0.407 | 0.01 |
| **Prostate Cancer (n=88)** | | | | | | |
|  | Unadjusted Model (***p* = 0.0244**) | | | Adjusted Model^b^ (***p* = 0.0194**) | | |
| As | <0.001 (0.006) | 0.962 | <0.001 | 0.002 (0.006) | 0.764 | 0.001 |
| Mn | 0.19 (0.10) | 0.062 | 0.04 | 0.19 (0.11) | 0.091 | 0.04 |
| Fe | 8.52 (4.56) | 0.065 | 0.04 | 8.09 (5.24) | 0.127 | 0.03 |
| Ni | 2.49 (3.71) | 0.504 | 0.005 | 3.18 (4.05) | 0.435 | 0.01 |
| Cu | 0.206 (0.232) | 0.377 | 0.01 | 0.02 (0.23) | 0.919 | <0.001 |
| Zn | 7.68 (2.98) | **0.012** | 0.07 | 7.70 (2.99) | **0.012** | 0.09 |
| Se | 0.06 (0.02) | **0.012** | 0.07 | 0.06 (0.02) | **0.011** | 0.09 |
| Cd | 0.002 (0.002) | 0.170 | 0.02 | 0.001 (0.002) | 0.531 | 0.003 |
| Pb | 0.05 (0.03) | 0.062 | 0.04 | 0.05 (0.03) | 0.089 | 0.04 |
| **Skin Cancer (n=86)** | | | | | | |
|  | Unadjusted Model (***p* = 0.0321**) | | | Adjusted Model^b^ (*p* = 0.4454) | | |
| As | -0.01 (0.005) | 0.067 | 0.04 | -0.01 (0.005) | 0.110 | 0.04 |
| Mn | 0.001 (0.09) | 0.987 | <0.001 | -0.02 (0.10) | 0.813 | 0.001 |
| Fe | -0.34 (4.26) | 0.936 | <0.001 | 0.10 (4.57) | 0.982 | <0.001 |
| Ni | -2.26 (3.73) | 0.546 | 0.004 | -0.66 (4.29) | 0.878 | <0.001 |
| Cu | 0.01 (0.20) | 0.953 | <0.001 | 0.01 (0.22) | 0.950 | <0.001 |
| Zn | 9.83 (2.92) | **0.001** | 0.12 | 11.57 (3.17) | **0.001** | 0.16 |
| Se | <0.001 (0.02) | 0.994 | <0.001 | -0.004 (0.03) | 0.873 | <0.001 |
| Cd | <\|0.001\| (0.002) | 0.941 | <0.001 | -0.0002 (0.001) | 0.908 | <0.001 |
| Pb | -0.01 (0.02) | 0.522 | 0.005 | -0.02 (0.02) | 0.334 | 0.01 |

^a^ Partial $\eta^{2}$ as a measure of effect size

^b^ Model adjusted for age, family history of cancer, province of residence, water source, physical activity, smoking status, and BMI
