# Supplementary material for: Toenail arsenic species and metallome profiles associated with breast, cervical, prostate, and skin cancer prevalence in the Atlantic Partnership for Tomorrow’s Health cohort
Source: Front Public Health. 2023 Jun 15;11:1148283. doi: 10.3389/fpubh.2023.1148283 (PMC10308375; doi:10.3389/fpubh.2023.1148283)
Supplement: Supplementary file 2 [file Data_Sheet_1.pdf]

Confidential

Supplemental Material: Detailed Model Input Parameters

Article: Public Health Impact and Return on Investment of Belgium's Pediatric Immunization Program

Table A-1. Summary of Pre-Vaccine and Vaccine-Era Disease Incidence Sources

Disease	Dates of vaccination program initiation[a]	Pre-vaccine sources (years of incidence data)	Vaccine-era sources (years of incidence data)
Diphtheria	1959	Agentschap Zorg en Gezondheid [1], Statbel and SPF Economie [2] (1950-1959)	Statbel and SPF Economie [2], ECDC [3] (2013-2017)
Hepatitis B	1999	Statbel and SPF Economie [2], ECDC [4][b] (2014-2018)	Statbel and SPF Economie [2], ECDC [4] (2014-2018)
Hib	2002	Reinert et al. [5] (1980-1989 [France])	Statbel and SPF Economie [2], ECDC [6] (2013-2017)
Measles	1985	Statbel and SPF Economie [2], Van Casteren [7] (1994-1995)	Statbel and SPF Economie [2], ECDC [8] (2015-2019)
Meningitis C	2002	Statbel and SPF Economie [2], Noah [9], ECDC [10] (1999-2002)	Statbel and SPF Economie [2], Jacquinet et al. [11] (2014-2018)
Mumps	1985	Statbel and SPF Economie [2], Van Casteren [7] (1994-1995)	Statbel and SPF Economie [2], ECDC [12] (2015-2019)
Pertussis	1959	Gałązka [13] (1961-1965 [Poland])	Statbel and SPF Economie [2], ECDC [14] (2013-2017)

Disease	Dates of vaccination program initiation[a]	Pre-vaccine sources (years of incidence data)	Vaccine-era sources (years of incidence data)
Polio	1959	Cockburn and Drozdov [15] (1951-1955)	Statbel and SPF Economie [2], ECDC [16] (2013-2017)
S pneumoniae	2007[c]		
Invasive pneumococcal disease		Mendes da Costa et al. [17], Blommaert et al. [18], Vergison et al. [19] (ages <18 years: 2002-2006; ages 18+: 2009-2011)	Mendes da Costa et al. [17], Braeye et al. [20] (2012-2016)
Pneumococcal hospitalizations		Beutels et al. [21] (2001-2004)	Mendes da Costa et al. [17], Braeye et al. [20], Beutels et al. [22] (2012-2016)
Pneumococcal outpatient visits		Beutels et al. [21] (2001-2004)	Mendes da Costa et al. [17], Blommaert et al. [18], Braeye et al. [20] (2012-2016)
Pneumococcal acute otitis media		Beutels et al. [21] (2002-2006)	Kawai et al. [23] (2012-2014 [United States])
Rotavirus	2006-2007	Jit et al. [24], Bilcke et al. [25], Bilcke et al. [26] (2002)	Sabbe et al. [27], Zeller et al. [28] (2010)
Rubella	1974	NIZP-PZH [29], NIZP-PZH [30], NIZP-PZH [31], NIZP-PZH [32], NIZP-PZH [33] (1999-2003 [Poland])	Statbel and SPF Economie [2], ECDC [34] (2015-2019)
Tetanus	1959	Statbel and SPF Economie [2], Kostrzewski [35] (1957-1961 [Poland])	Statbel and SPF Economie [2], ECDC [36] (2013-2017)

ECDC = European Centre for Disease Prevention and Control; Hib = *Haemophilus influenzae type b*; PCV-7 = pneumococcal conjugate vaccine (7-valent); PCV-13 = pneumococcal conjugate vaccine (13- valent).

[a] Dates of vaccination program initiation correspond to dates of vaccine licensure and/or routine use.

[b] Reporting of hepatitis B incidence improved after the introduction of hepatitis B vaccination, and thus pre-vaccine incidence is likely significantly underreported and the degree of underreporting is not known. Therefore, pre-vaccine incidence was assumed to be the same as vaccine-era incidence.

[c] Date corresponds to introduction of PCV-7. As of 2019, Belgium's Superior Health Council recommends routine immunization with PCV-13.

Table A-2. Incidence of Adverse Events per 100,000 Doses

	Incidence of adverse events per 100,000 doses, by vaccine					
**Adverse event**	**DTaP-IPV**	**MMR**	**PCV**	**Rotavirus**	**Hexavalent**	**MenC**
Anaphylaxis	0.1	0.1	0.1	-	0.1	0.1
Arthralgia	-	1,000	-	-	-	-
Aseptic meningitis	-	0.1	-	-	-	-
Vaccine-related encephalitis	-	0.1	-	-	-	-
Injection site reaction (severe)	-	2,000	2,000	-	-	-
Intussusception	-	-	-	1.0	-	-
Mild gastroenteritis/vomiting	-	-	-	225	-	-
Parotitis	-	1,600	-	-	-	-
Seizure	-	33	0.5	-	-	-
Thrombocytopenic purpura	-	3	-	-	-	-
AE outpatient visit	5,000	-	-	-	5,000	5,000

DTaP = diphtheria, tetanus, and acellular pertussis; HepB = hepatitis B; Hib = *Haemophilus influenzae type b*; IPV = inactivated polio vaccine; MenC = Meningitis C; MMR = measles, mumps, rubella; PCV = pneumococcal conjugate vaccine.

Sources: Default adverse event incidence data were obtained from Zhou et al. [37] for MMR; Zhou et al. [38] for PCV; Widdowson et al. [39], Zhou et al. [38], and Bilcke et al. [40] for rotavirus; and Hanquet et al. [41] for DTaP-IPV, hexavalent, and MenC vaccines. A rate of one anaphylaxis event per 1,000,000 doses was assumed for all injected vaccines [42-44]. Two percent of doses for MMR and PCV were assumed to cause severe injection site reactions per expert opinion and review of package inserts for pediatric vaccines.

Table A-3. Costs and QALY Losses per Adverse Event

Adverse event (vaccine)	Cost per adverse event[a]	QALY loss per adverse event	Sources and assumptions
Anaphylaxis (all injected vaccines)	€1,451	0.0200	The default cost for anaphylaxis was calculated based on the resource use from previous economic analyses of childhood vaccination [45]. This previous study assumed that 100% of anaphylaxis patients were hospitalized with a mean 2.89 hospital days. The analysis assumes patients have 2.39 outpatient visits, based on a calculation that divided the cost of outpatient visits for anaphylaxis by a proxy for the cost of one visit [45]. The default QALY loss for anaphylaxis was obtained from Kamiya et al. [46], citing Lee et al. [47].
Arthralgia (MMR)	€65	0.0001	Based on resource use from a previous economic analysis of childhood vaccination which assumed that 1% of patients were hospitalized with a mean of 2 hospital days [45]. The analysis assumed patients have 2.19 outpatient visits, based on a calculation that divided the cost of outpatient visits for arthralgia by a proxy for the cost of one visit [45]. Assumed same QALY loss as mild complication, which was obtained from Tu et al. [48].
Aseptic meningitis (MMR)	€418	0.0232	Based on resource use from a previous economic analysis of childhood vaccination which assumed that 25% of patients were hospitalized with a mean of 3 hospital days [45]. The analysis assumed patients have 3.24 outpatient visits, based on a calculation that divided the cost of outpatient visits for aseptic meningitis by a proxy for the cost of one visit [45]. The default disutility for aseptic meningitis was assumed to be the same as the utility loss for meningitis due to invasive pneumococcal disease, which was reported in Rubin et al. [49].

Adverse event (vaccine)	Cost per adverse event[a]	QALY loss per adverse event	Sources and assumptions
Vaccine-related encephalitis (MMR)	€4,320	0.0534	Based on resource use from a previous economic analysis of childhood vaccination which assumed that 100% of patients with encephalitis due to MMR were hospitalized with a mean of 8.7 hospital days [45]. The analysis assumed patients have 5.28 outpatient visits, based on a calculation that divided the cost of outpatient visits for arthralgia by a proxy for the cost of one visit [45]. The default QALY loss was calculated by adding the QALY loss for acute encephalitis and for encephalitis that results in lifetime disability. The QALY for acute encephalitis was obtained from Anyiwe et al. [50] which assumed a disutility value of 0.21 for a 2-week duration, resulting in a QALY loss of 0.01. The disutility for encephalitis that results in lifetime disability was obtained from Philipson et al. [51] which assumed a disutility of 0.56. The model assumed that this disutility would occur for the remaining 50 weeks of the year, resulting in a QALY loss of 0.54. Fowler et al. [52] reported that 8.5% of encephalitis cases result in long-term disability. This percentage was applied to the QALY loss for encephalitis resulting in long-term disability and then added to the QALY loss for acute encephalitis to calculate the assumed QALY loss for vaccine-related encephalitis.
Injection site reaction (severe) (MMR, PCV)	€25	0.0014	The analysis assumed that patients have 1 outpatient visit [45]. The default disutility and duration were assumed to be 0.5 for 1 day per expert opinion, which is similar to QALY loss for injection site reaction for Tdap vaccine in adolescents (0.001) [46].
Intussusception (rotavirus)	€0	0.0232	The default QALY loss was assumed the same as aseptic meningitis [49]. No cost was assumed because the vaccine has been designated safe [40].
Mild gastroenteritis/vomiting (rotavirus)	€0	0.0006	The default disutility for mild gastroenteritis/vomiting of 0.219 was based on the disutility for non-hospitalized diarrhea [53]. The default duration applied was 1-day per expert opinion. No cost was assumed because the vaccine has been designated safe [40].

Adverse event (vaccine)	Cost per adverse event[a]	QALY loss per adverse event	Sources and assumptions
Parotitis (MMR)	€64	0.0002	Based on resource use from a previous economic analysis of childhood vaccination which assumed that 1% of patients were hospitalized with a mean of 3 hospital days. The analysis assumed patients have 1.94 outpatient visits, based on a calculation that divided the cost of outpatient visits for parotitis by a proxy for the cost of one visit [45]. Hospitalization cost per day was obtained from and outpatient visit cost was obtained from. The default QALY loss for parotitis was assumed to be slightly higher than mild complication per expert opinion.
Seizure (MMR, PCV)	€263	0.0004	Based on resource use from a previous economic analysis of childhood vaccination which assumed that 10% of patients were hospitalized with a mean of 2 hospital days [45]. The analysis assumed patients have 6.55 outpatient visits, based on a calculation that divided the cost of outpatient visits for parotitis by a proxy for the cost of one visit [45]. The default QALY loss for a seizure was obtained from Tu et al. [48].
Thrombocytopenic purpura (MMR)	€1,090	0.0232	Based on resource use from a previous economic analysis of childhood vaccination which assumed that 40% of patients were hospitalized with a mean of 4.8 hospital days [45]. The analysis assumed patients have 6.52 outpatient visits, based on a calculation that divided the cost of outpatient visits for parotitis by a proxy for the cost of one visit [45]. The default QALY loss for thrombocytopenic purpura was assumed to be the same as aseptic meningitis [49].
Adverse event outpatient visit (DTaP-IPV, Hexavalent, MenC)	€25	0.0001	The analysis assumed that patients have 1 outpatient visit [45]. The default QALY loss for adverse event outpatient visit was assumed to be the same as mild complication [48].

DTaP = diphtheria, tetanus, and acellular pertussis; MMR = measles, mumps, rubella; MenC = Meningitis CPCV = pneumococcal conjugate vaccine; QALY = quality-adjusted life-year.

[a] Hospitalization cost per day (€481.17) was obtained from Cleemput et al. [54] and was inflated from 2012 to 2020 Belgian Euros using the Gross Domestic Product deflator index from the CCEMG - EPPI-Centre Cost Converter. The outpatient visit cost (€25.44) is the average cost of a general practitioner and pediatrician visit from RIZIV/INAMI [55].

Table A-4. Diphtheria Case Severity Distribution, Disease-Related Deaths, and Direct Medical Costs per Case

Case severity	Case severity distribution	Sources	Cost per case	Sources
Hospitalized case	100.0%	Carrico et al. [56], citing Ekwueme et al. [57]	€2,935.14	Hospitalization cost calculated based on the length of stay of 6.1 days [58] and cost per day from Cleemput et al. [54][a]
Outpatient case	0.0%		N/A	
Percentage of medically attended cases resulting in death	10.0%	Carrico et al. [56], citing Zhou et al. [45]	N/A	

CPI = consumer price index; N/A = not applicable.

[a] Inflated to 2020 Belgian Euros using the Belgian CPI healthcare inflation index [59].

Table A-5. Hepatitis B Case Severity Distribution, Disease-Related Deaths, and Direct Medical Costs per Case

Case severity	Case severity distribution <1 mo	1 mo-1 y	1-4 y	5-14 y	≥15 y	Sources	Cost per case	Sources
Acute symptomatic	1.0%	6.0%	6.0%	30.0%	30.0%	Carrico et al. [56], citing Zhou et al. [45], citing Margolis et al. [60]		
Fulminant case	0.1%	0.1%	0.6%	0.6%	0.6%		€4,292	Tormans et al. [61][a]
Hospitalized case	62.7%	62.7%	62.2%	62.2%	62.2%		€1,989	
Outpatient case (jaundice)	37.2%	37.2%	37.2%	37.2%	37.2%		€402	Miriti et al. [62][b]
Acute asymptomatic	99.0%	94.0%	94.0%	70.0%	70.0%		€0	
Percentage of cases resulting in long-term sequelae								
Chronic HepB (among acute cases)	90.0%	90.0%	30.0%	6.0%	6.0%	Carrico et al. [56], citing CDC [63]		
Liver transplant (among fulminant cases)	12.0%	12.0%	12.0%	12.0%	12.0%	Carrico et al. [56], citing Miriti et al. [62]	€120,757 (year of transplant); €9,238 (annual cost remaining lifetime)	Gerkens et al. [64][a]

Case severity	Case severity distribution <1 mo	1 mo-1 y	1-4 y	5-14 y	≥15 y	Sources	Cost per case	Sources
Percentage of fulminant cases resulting in death	70.0%	70.0%	70.0%	70.0%	70.0%	Carrico et al. [56], citing Zhou et al. [45], citing Mulley et al. [65], Bloom et al. [66], Margolis et al. [60], Arevalo and Washington [67], and Krahn and Detsky [68]		
Annual transitions from chronic HepB and associated complications								
Chronic HepB						Carrico et al. [56], citing Miriti et al. [62]		
Compensated cirrhosis	0.5%	0.5%	0.5%	0.5%	0.5%			
HCC	0.02%	0.02%	0.02%	0.02%	0.02%			
Compensated cirrhosis							€1,393	Gerkens et al. [64][a]
Decompensated cirrhosis	3.4%	3.4%	3.4%	3.4%	3.4%			
HCC	2.5%	2.5%	2.5%	2.5%	2.5%			
Death	3.8%	3.8%	3.8%	3.8%	3.8%			
Decompensated cirrhosis							€8,310	Gerkens et al. [64][a]
HCC	2.5%	2.5%	2.5%	2.5%	2.5%			

Case severity	Case severity distribution <1 mo	1 mo-1 y	1-4 y	5-14 y	≥15 y	Sources	Cost per case	Sources
Liver transplant	1.0%	1.0%	1.0%	1.0%	1.0%			
Death	26.4%	26.4%	26.4%	26.4%	26.4%			
HCC							€13,517	Gerkens et al. [64][a]
Liver transplant	2.0%	2.0%	2.0%	2.0%	2.0%			
Death	71.1%	71.1%	71.1%	71.1%	71.1%			
Annual probability of hospitalization for acute exacerbation among those with chronic HepB	0.1%	0.1%	0.1%	0.1%	0.1%		€2,550	Tormans et al. [61][a]
Percentage of liver transplants resulting in death								
Year of transplant	11.6%	11.6%	11.6%	11.6%	11.6%	Carrico et al. [56], citing Dhankhar et al. [69], citing Wolfe et al. [70]		
Subsequent years	4.4%	4.4%	4.4%	4.4%	4.4%			

CDC = Centers for Disease Control and Prevention; CPI = consumer price index; HCC = hepatocellular carcinoma; HepB = hepatitis B; US = United States.

[a] Inflated to 2020 Belgian Euros using the Belgian CPI healthcare inflation index [59].

[b] Inflated to 2020 US dollars using US PCE healthcare inflation factor [71]. Costs were then adjusted to 2020 Belgian Euros using the Gross Domestic Product deflator index from the CCEMG - EPPI-Centre Cost Converter.

Table A-6. Hib Case Severity Distribution, Disease-Related Deaths, and Direct Medical Costs per Case

	Case severity distribution								
	Pre-vaccine			Vaccine era					
Case severity	0-5 mo	6-11 mo	≥1 y	0-5 mo	6-11 mo	≥1 y	Sources	Cost per case	Sources
Meningitis case	68.2%	65.1%	54.6%	39.6%	54.9%	32.3%	Carrico et al. [56], citing Zhou et al. [72]	€6,197-€10,745[a]	Blommaert et al. [18], Phoenix et al. [73], Garpenholt et al. [74][b]
Epiglottitis case	0.7%	0.7%	19.4%	0.0%	0.0%	3.2%		€2,395	
Bacteremia case	8.6%	9.5%	6.7%	32.2%	18.5%	29.8%		€3,167-€9,174[a]	
Pneumonia case	9.6%	10.5%	7.5%	7.9%	2.8%	9.2%		€3,951-€8,077[a]	
Cellulitis case	10.5%	11.6%	8.2%	2.3%	2.8%	5.7%		€2,480	
Arthritis case	1.6%	1.7%	2.4%	0.0%	1.4%	0.4%		€3,508	
Other invasive case	0.8%	0.9%	1.2%	18.1%	19.7%	19.4%		€3,784-€6,040[a]	
Percentage of Hib meningitis cases resulting in long-term sequelae									Blommaert et al. [18][b]
Major cognitive difficulties	9.3%	9.3%	9.3%	9.3%	9.3%	9.3%	Carrico et al. [56], citing Christie et al. [75]	€38,983 (annual cost for 50 y)	
Major hearing loss	8.2%	8.2%	8.2%	8.2%	8.2%	8.2%	Carrico et al. [56], citing Jit [76]	€42,821 (special education cost applied from ages 3-18 y); €4,916 (one-time cost)	

	**Case severity distribution**								
	**Pre-vaccine**			**Vaccine era**					
**Case severity**	**0-5 mo**	**6-11 mo**	**≥1 y**	**0-5 mo**	**6-11 mo**	**≥1 y**	**Sources**	**Cost per case**	**Sources**
Percentage of Hib cases resulting in death	3.8%	3.8%	3.8%	3.8%	3.8%	3.8%	Carrico et al. [56], citing Zhou et al. [72], citing Cochi et al. [77]	N/A	

CPI = consumer price index; Hib = *Haemophilus influenzae* type b; NA = not applicable.

[a] Cost varies based on age group.

[b] Inflated to 2020 Belgian Euros using the Belgian CPI healthcare inflation index [59].

Table A-7. Measles Case Severity Distribution, Disease-Related Deaths, and Direct Medical Costs per Case

	Case severity distribution						Cost per case					
**Case severity**	**<1 y**	**1 y**	**2-4 y**	**5-19 y**	**≥20 y**	**Sources**	**<1 y**	**1 y**	**2-4 y**	**5-19 y**	**≥20 y**	**Sources**
Encephalitis case	0.1 %	0.1 %	0.1 %	0.1 %	0.2 %	Carrico et al. [56], citing Zhou et al. [37]	€172,610	€160,708	€146,911	€130,917	€65,959	Beutels et al. [78][a]
Pneumonia case	7.0 %	7.2 %	5.2 %	1.6 %	4.7 %		€484	€541	€572	€572	€572	
Otitis media case	12.0 %	13.5 %	8.7 %	2.2 %	1.4 %		€157	€180	€192	€192	€192	
Uncomplicated or diarrhea case	81.0 %	79.2 %	86.0 %	96.1 %	1.4 %		€85	€99	€102	€107	€130	
Percentage of encephalitis cases resulting in long-term disability	8.5 %	8.5 %	8.5 %	8.5 %	8.5 %	Carrico et al. [56], citing Fowler et al. [52]			NA[b]			
Percentage of reported cases resulting in death	0.08 %	0.08 %	0.08 %	0.08 %	0.08 %	Carrico et al. [56], citing Zhou et al. [37], citing Pelletier et al. [79]	N/A	N/A	N/A	N/A	N/A	

CPI = consumer price index; NA = not applicable.

[a] Inflated to 2020 Belgian Euros using the Belgian CPI healthcare inflation index [59].

[b] The annual cost of long-term disability due to encephalitis is included in the direct cost per case of measles encephalitis.

Table A-8. MenC Case Severity Distribution, Disease-Related Deaths, and Direct Medical Costs per Case

Case severity	Case severity distribution	Sources	Cost per case					Sources
			<1 y	1-4 y	5-9 y	10-19 y	≥20 y	
Hospitalized case	74.6%	Jacquinet et al. [11]	€7,797.81	€6,768.65	€6,456.78	€8,619.80	€11,456.69	Hanquet et al. [41][a]
Outpatient visit	25.4%		€108.20	€108.20	€108.20	€108.20	€108.20	
Percentage of cases resulting in death								
Pre-vaccine	7.1%-35.7%[b]	Hanquet et al. [41]	N/A	N/A	N/A	N/A	N/A	
Vaccine era	0.0%	Jacquinet et al. [11]	N/A	N/A	N/A	N/A	N/A	

Parameter	Percentage resulting in long-term sequelae: Age Group (y) <20	Percentage resulting in long-term sequelae: Age Group (y) ≥20	Sources	Annual costs per long-term sequelae: One-time cost <18	One-time cost ≥18	Recurring cost[c] <18	Recurring cost[c] ≥18	Sources
Severe hearing loss	0.8%	0.4%	Hanquet et al. [41]	€1,179.53	€589.21	€629.30	€377.58	Hanquet et al. [41][a]
Neurological sequelae	1.1%	0.5%		€0.00	€0.00	€46,379.20	€46,379.20	
Skin graft	1.6%	3.7%		€2,519.45	€2,519.45	€0.00	€0.00	
Epilepsy	4.7%	0.0%		€0.00	€0.00	€270.66	€530.18	
Renal failure	0.4%	3.7%		€5,735.03	€5,735.03	€0.00	€0.00	
Minor amputation	0.3%	1.0%		€4,559.95	€4,559.95	€1,515.90	€302.96	
Major amputation (bilateral)	0.3%	0.4%		€28,353.24	€28,353.24	€8,124.16	€2,321.19	

CPI = Consumer Price Index; MenC = Meningitis C; NA = not applicable.

Note:

[a] Inflated to 2020 Belgian Euros using the Belgian CPI healthcare inflation index [59].

[b] Percentage of cases resulting in death varies by age group.

[c] Recurring costs are applied each year of individual's remaining lifetime.

Table A-9. Mumps Case Severity Distribution, Disease-Related Deaths, and Direct Medical Costs per Case

	Case severity distribution			Cost per case		
**Case severity**	**<15 y**	**≥15 y**	**Sources**	**<15 y**	**≥15 y**	**Sources**
Complicated case	11.5%	48.5%	Carrico et al. [56], citing Zhou et al. [37]	€4,900.69	€1,541.16	Zhou et al. [37][a]
Uncomplicated case	88.5%	51.5%		€126.61	€126.61	
Percentage of reported cases resulting in death	0.0%	0.0%	Sciensano [80]	N/A	N/A	

NA = not applicable; US = United States.

[a] Inflated to 2020 US dollars using US PCE healthcare inflation factor [71]. Costs were then adjusted to 2020 Belgian Euros using the Gross Domestic Product deflator index from the CCEMG - EPPI-Centre Cost Converter.

Table A-10. Pertussis Case Severity Distribution, Disease-Related Deaths, and Costs per Case

Case severity	Case severity distribution	Sources	Cost per case			Sources
			<1 y	1-19 y	≥20 y	
Hospitalized case	7.8%	ECDC [12]	€3,414.07	€976.15	€976.15	Calculated from de Greeff et al. [81] and RIZIV/INAMI [55]
Outpatient case	92.2%		€25.90	€26.23	€21.38	
Percentage of medically attended cases resulting in death	0.04%	Carrico et al. [56], citing CDC [82]	N/A	N/A	N/A	

ECDC = European Centre for Disease Prevention and Control; CDC = Centers of Disease Control and Prevention; NA = not applicable.

Table A-11. Pneumococcal Disease Case Severity Distribution, Disease-Related Deaths, and Direct Medical Costs per Case

Parameter	Age groups (y)										Sources
	<1	1-4	5-9	10-14	15-19	20-24	25-44	45-64	65-74	75+	
Case severity[a]											
Acute otitis media case											
Hospitalized case	2.5%	1.7%	1.7%	0.3%	0.3%	0.2%	0.3%	0.1%	0.4%	0.7%	Beutels et al. [21]
Non-hospitalized case	97.5%	98.3%	98.3%	99.7%	99.7%	99.8%	99.7%	99.9%	99.6%	99.3%	
Percentage of meningitis cases resulting in long-term sequelae											
Major cognitive difficulties	12.9%	12.9%	12.9%	12.9%	12.9%	12.9%	12.9%	12.9%	12.9%	12.9%	Willem et al. [83]
Major hearing loss	12.9%	12.9%	12.9%	12.9%	12.9%	12.9%	12.9%	12.9%	12.9%	12.9%	
**Parameter**	**Age Groups (y)**										
	**<1**	**1-4**	**5-9**	**10-14**	**15-19**	**18-49**	**50-64**	**65-74**	**75-84**	**85+**	**Sources**
Percentage of cases resulting in death											
IPD case (meningitis or bacteremia)	3.4%	1.9%	3.0%	0.0%	4.7%	6.7%	16.1%	15.8%	18.6%	23.4%	Blommaert et al. [18]
Hospitalized pneumococcal pneumonia	0.3%	0.2%	0.7%	1.7%	2.4%	3.5%	4.9%	5.5%	6.7%	8.2%	Beutels et al. [21]

**Parameter**	**Age Groups (y)**						
	**<5**	**5-17**	**18-34**	**35-49**	**50-64**	**≥65**	**Sources**
Cost per case							
IPD							<18: Beutels et al. [21][b] ≥18: Blommaert et al. [18][b]
Hospitalized case - meningitis	€10,744.58	€10,605.04	€6,033.30	€6,033.30	€8,334.28	€9,402.81	
Hospitalized case - bacteremia	€3,166.89	€9,173.76	€4,060.88	€4,060.88	€8,798.38	€ 5,873.84	
Pneumococcal pneumonia							
Hospitalization	€4,933.07	€7,129.83	€3,758.34	€3,758.34	€6,147.16	€5,526.71	
Outpatient visit	€947.54	€947.54	€87.72	€87.72	€87.72	€87.72	
Acute otitis media							Beutels et al. [21] [b]
Complex case	€4,082.54	€4,552.99	€4,552.99	€4,552.99	€4,552.99	€4,552.99	
Simple case	€371.44	€91.03	€91.03	€91.03	€91.03	€91.03	

CPI = consumer price index; IPD = invasive pneumococcal disease.

[a] Case severity distribution for acute otitis media, IPD, and pneumococcal pneumonia determined from disease incidence data.

[b] Inflated to 2020 Belgian Euros using the Belgian CPI healthcare inflation index [59].

Table A-12. Polio Case Severity Distribution, Disease-Related Deaths, and Direct Medical Costs per Case

Case severity	Case severity distribution	Sources	Cost per case	Sources
Paralytic case	100.0%	Carrico et al. [56], citing Roush and Murphy [84] and CDC [85]	€88,713.79	Chan et al. [86][a]
Nonparalytic case	0.0%		$0.00	CDC [87]
Percentage of paralytic cases resulting in permanent paralysis	0.5%	Carrico et al. [56], citing WHO [88]	€11,599.82 (annual cost for remaining lifetime)	Chan et al. [86][a]
Percentage of paralytic cases resulting in death	2.0%	Carrico et al. [56], citing CDC [87]	N/A	

CDC = Centers for Disease Control and Prevention; CPI = consumer price index; NA = not applicable; WHO = World Health Organization.

[a] Inflated to 2020 Belgian Euros using the Belgian CPI healthcare inflation index [59].

Table A-13. Rotavirus Case Severity Distribution, Disease-Related Deaths, and Direct Medical Costs per Case

Case severity[a]	Case severity distribution				Sources	Cost per case[b]	Sources
	Pre-vaccine		Vaccine era				
	<1 y	1-4 y	<1 y	1-4 y			
Hospitalization	36.4%	15.4%	26.5%	14.7%	Bilcke et al. [25], Bilcke et al. [26], Sabbe et al. [27], Zeller et al. [28], Jit [76]	€1,063.19	Bilcke et al. [26]
ED visit	0.0%	0.0%	70.5%	0.0%		€0.00[c]	
Outpatient visit	60.9%	81.1%	0.0%	81.8%		€106.59	
NMA case	2.6%	3.5%	3.0%	3.5%		€21.73	
Percentage of medically attended cases resulting in death	0.0043%		0.0043%		Carrico et al. [56], citing Widdowson et al. [39]	N/A	

CPI = consumer price index; ED = emergency department; NA = not applicable; NMA = non–medically attended.

[a] Case severity for rotavirus determined from incidence rates for each severity level.

[b] Inflated to 2020 Belgian Euros using the Belgian CPI healthcare inflation index [59].

[c] Cost of ED visits is set to €0 as Bilcke et al. [26] reports that a majority of hospitalized children are admitted via the ED (74%).

Table A-14. Rubella Case Severity Distribution, Disease-Related Deaths, and Direct Medical Costs per Case

	Case severity distribution								
	Pre-vaccine			Vaccine era					
Case severity	<1 y	1-14 y	≥15 y	<1 y	1-14 y	≥15 y	Sources	Cost Per Case	Sources
CRS	0.17%	0.00%	0.00%	88.89%	0.00%	0.00%	Pre-vaccine: Carrico et al. [56], citing Roush and Murphy [84]; Vaccine era: Carrico et al. [56], citing CDC [89], CDC [90], CDC [91], Adams et al. [92], Adams et al. [93]	€40,946.55	Lugner et al. [94][a]
Complicated case	0.04%	0.04%	30.05%	0.04%	0.04%	30.05%	Carrico et al. [56], citing Zhou et al. [37]	€40,946.55	
Uncomplicated case	99.79%	99.96%	69.96%	11.07%	99.96%	69.96%		€51.63	
Percentage of CRS cases resulting in long-term sequelae									
Mental retardation – moderate	27.1%	27.1%	27.1%	27.1%	27.1%	27.1%	Carrico et al. [56], citing Zhou et al. [37]	€38,774.67 (annual cost for 50-year duration)	[a]

	Case severity distribution								
	Pre-vaccine			Vaccine era					
**Case severity**	**<1 y**	**1-14 y**	**≥15 y**	**<1 y**	**1-14 y**	**≥15 y**	**Sources**	**Cost Per Case**	**Sources**
Mental retardation – severe	5.9%	5.9%	5.9%	5.9%	5.9%	5.9%		€102,442.24 (annual cost for 50-year duration)	
Learning disabilities	47.0%	47.0%	47.0%	47.0%	47.0%	47.0%		€20,910.96 (annual cost applied from age 3-18 y)	RIZIV/INAMI [55]
Percentage of cases resulting in death									
CRS (first year)	10.600%	10.600%	10.600%	10.600%	10.600%	10.600%	Carrico et al. [56], citing Zhou et al. [37]	N/A	
CRS (second year)	0.400%	0.400%	0.400%	0.400%	0.400%	0.400%		N/A	
Non-congenital rubella	0.001%	0.001%	0.001%	0.001%	0.001%	0.001%		N/A	

CDC = Centers for Disease Control and Prevention; CPI = consumer price index; CRS = congenital rubella syndrome; NA = not applicable; US = United States.

[a] Inflated to 2020 Belgian Euros using the Belgian CPI healthcare inflation index [59].

[b] Inflated to 2020 US dollars using US PCE healthcare inflation factor [71]. Costs were then adjusted to 2020 Belgian Euros using the Gross Domestic Product deflator index from the CCEMG - EPPI-Centre Cost Converter.

Table A-15. Tetanus Case Severity Distribution, Disease-Related Deaths, and Direct Medical Costs per Case

Case severity	Case severity distribution	Sources	Cost per Case	Sources
Hospitalized case	100.0%	Carrico et al. [56], citing Ekwueme et al. [57]	€76,988.44	Ekwueme et al. [57], citing Hatziandreu et al. [95][a]
Outpatient visit	0.0%		N/A	
Percentage of medically attended cases resulting in death	15.0%	Carrico et al. [56], citing Zhou et al. [45]	N/A	

NA = not applicable; NMA = non–medically attended; US = United States.

[a] Inflated to 2020 US dollars using US PCE healthcare inflation factor [71]. Costs were then adjusted to 2020 Belgian Euros using the Gross Domestic Product deflator index from the CCEMG - EPPI-Centre Cost Converter.

Table A-16. Quality-of-Life Inputs

Disease	Disutility	Duration of disutility (years)	QALY loss	Source
Diphtheria	0.23	0.03	0.006	Philipson et al. [51], citing WHO [96]; CDC [97]
HepB				
Acute case	0.29	0.02	0.006	Chahal et al. [98]
Fulminant case	0.62	0.04	0.024	
Chronic HepB	0.14	Remaining lifetime (or until transition)	0.14 per year	
Compensated cirrhosis	0.33		0.33 per year	
Decompensated cirrhosis	0.62		0.620 per year	
Hepatocellular carcinoma	0.56		0.560 per year	
Liver transplant	0.35	Remaining lifetime	0.350 per year	
Hib				
Hib infection	0.62	0.03	0.017	Philipson et al. [51], citing WHO [96]; CDC [99]
Major cognitive difficulties	0.38	Remaining lifetime	0.380 per year	Oostenbrink et al. [100]
Major hearing loss	0.09	Remaining lifetime	0.090 per year	Oostenbrink et al. [100]
Measles				
Measles case	0.15	0.04	0.006	Philipson et al. [51], citing WHO [96]; CDC [87]
Long-term disability due to encephalitis	0.56	50.00	0.560 per year	
Mumps	0.15	0.03	0.004	Philipson et al. [51], citing WHO [96], National Health Service [101]
Pertussis	0.19	0.08	0.015	Philipson et al. [51], citing Greer and Fisman [102], CDC [87]
Pneumococcal disease				

Disease	Disutility	Duration of disutility (years)	QALY loss	Source
IPD meningitis	0.023	1.0	0.023	Rubin et al. [49], citing Bennett et al. [103] and Oh et al. [104]
IPD bacteremia	0.008	1.0	0.008	
All-cause pneumonia hospitalizations	0.006	1.0	0.006	
All-cause pneumonia outpatient visits	0.004	1.0	0.004	
All-cause acute otitis media	0.005	1.0	0.005	
Major cognitive difficulties	0.380	Remaining lifetime	0.380 per year	Oostenbrink et al. [100]
Major hearing loss	0.090	Remaining lifetime	0.090 per year	Oostenbrink et al. [100]
Polio				
Paralytic polio	0.369	0.50	0.185	Khan and Ehreth [105]; Harvard Medical School [106]
Permanent paralysis	0.369	Remaining lifetime	0.369 per year	Khan and Ehreth [105]
Nonparalytic polio	0.001	1.00	0.001	Prosser et al. [107]
Rotavirus				
Hospitalization	0.58	0.01	0.003	Martin et al. [53], citing Martin et al. [108]
ED visit	0.22	0.02	0.004	
Outpatient visit	0.22	0.02	0.004	
Non–medically attended case	0.22	0.01	0.003	
Rubella				
Rubella infection	0.15	0.02	0.003	Philipson et al. [51], citing WHO [96]; National Health Service [109]
CRS	0.35	Remaining lifetime	0.350 per year	Philipson et al. [51], citing WHO [96]
Tetanus	0.64	0.08	0.049	Philipson et al. [51], citing WHO [96], CDC [87]
MenC				

Disease	Disutility	Duration of disutility (years)	QALY loss	Source
Severe hearing loss	0.30	Remaining lifetime	0.30 per year	Hanquet et al. [41]
Neurological sequelae		Remaining lifetime	0.30 per year	
Skin graft		1.00	0.30	
Epilepsy		Remaining lifetime	0.30 per year	
Renal failure		1.00	0.30	
Minor amputation		Remaining lifetime	0.30 per year	
Major amputation (bilateral)		Remaining lifetime	0.30 per year	

CDC = Centers for Disease Control and Prevention; HepB = hepatitis B; Hib = *Haemophilus influenzae* type b; MenC = Meningitis C; WHO = World Health Organization.

Table A-17. Productivity Loss per Case, by Disease

Disease	Productivity lost per case (days)	Sources and assumptions
Diphtheria	6.1	Carrico et al. [56], citing Zhou et al. [38]
Tetanus	16.7	Carrico et al. [56], citing Zhou et al. [38]
Pertussis		
Hospitalized	10.3	Carrico et al. [56], citing Zhou et al. [38]
Outpatient	1.0	Assumption[a]
NMA	0.5	Assumption[a]
HepB		
Hospitalized (including fulminant)	33.2	MSD data on file (2020); based on data from Berge et al. [110]
Outpatient	15.5	
Hib		Carrico et al. [56], citing Zhou et al. [72]; productivity lost for hospitalized cases was assumed to be equal to the length of hospitalization (days) reported in Zhou et al. [72]; non-hospitalized cases were assumed to incur one caregiver day of time lost; an average number of caregiver days lost per Hib outcome was calculated based on the hospitalization rate for Hib outcomes reported in Zhou et al. [72].
Meningitis	7.2	
Epiglottitis	4.3	
Bacteremia	3.0	
Pneumonia	4.8	
Cellulitis	2.1	
Arthritis	1.5	
Other invasive disease	3.2	
Measles		Carrico et al. [56], citing Zhou et al. [37]
Encephalitis	8.7	
Pneumonia	4.5	
Otitis media	4.0	
Uncomplicated or diarrhea case	3.5	
MenC	1.0	Assumption[a]
Mumps		Carrico et al. [56], citing Zhou et al. [37]
Complicated	5.6	
Uncomplicated	4.0	
Rubella		Carrico et al. [56], citing Zhou et al. [37]
Complicated	5.1	
Uncomplicated	2.8	
Pneumococcal disease[a]		
IPD Meningitis	10.6	Carrico et al. [56], citing Lieu et al. [111]

Disease	Productivity lost per case (days)	Sources and assumptions
IPD Bacteremia	2.2	Carrico et al. [56], citing Lieu et al. [111]
Hospitalized pneumococcal pneumonia	1.9	Carrico et al. [56], citing Lieu et al. [111]
Outpatient pneumococcal pneumonia	1.0	Assumption[a]
AOM - complex	4.4	Carrico et al. [56], citing Lieu et al. [111]
AOM - simple	1.0	Carrico et al. [56]; estimated from Lieu et al. [111] and Ray et al. [112]
Polio		
Paralytic	182.6	Carrico et al. [56]; assumed 6 months of caregiver time loss based on Harvard Medical School [106] estimate of 6 months to 2 years for return of motor function
Nonparalytic	0.5	Carrico et al. [56]; assumed 0.5 days of work loss based on CDC [113] estimate that about 25% of cases of polio result in flu-like symptoms for 2-5 days
Rotavirus[b]		
Hospitalized	2.5	Bilcke et al. [26] for hospitalized and outpatient rotavirus; Carrico et al. [56], citing Mast et al. [114] for ED and NMA cases
ED	1.8	
Outpatient	1.5	
NMA	1.0	Carrico et al. [56], citing Widdowson et al. [39]

AOM = acute otitis media; ED = emergency department; HCC = hepatocellular carcinoma; HepB = hepatitis B; Hib = *Haemophilus influenzae* type b; IPD = invasive pneumococcal disease MenC = Meningitis C; NMA = non–medically attended.

[a] When no data were available to inform the estimate for days of productivity loss per case, we conservatively assumed one day of productivity loss for medically attended cases and 0.5 days for non–medically attended cases.

Table A-18. Percent Reduction in Annual Productivity for Long-Term Complications

Disease	Reduction in annual productivity
HepB	
Chronic HepB	14.0%
Compensated cirrhosis	33.0%
Decompensated cirrhosis	62.0%
HCC	56.0%
Liver transplant	35.0%
Hib	
Major cognitive difficulties	38.0%
Major hearing loss	9.0%
Measles	
Long-term disability due to encephalitis	56.0%
Meningitis C	
Long-term sequelae	30.0%
Pneumococcal	
Major cognitive difficulties	38.0%
Major hearing loss	9.0%
Polio	
Permanent paralysis	36.9%
Rubella	
CRS	35.0%

CRS = congenital rubella syndrome; HCC = hepatocellular carcinoma; HepB = hepatitis B; Hib = *Haemophilus influenzae* type b.

Note: Long-term complications were assumed to incur a percentage reduction in annual caregiver or patient productivity for patients’ remaining lifetime or the duration of the complication. The percentage reduction in annual productivity values were assumed to be equal to the disutility (i.e., percentage reduction in health-related quality-of-life) values applied for each long-term complication.

Table A-19. Market Productivity, by Age Group

Age group	Annual productivity[a]	Daily productivity[b]	Sources and assumptions
0-14 y	$0	€154.03	Annual age-specific market productivity (for value of time lost from work) estimates were obtained from Statbel and SPF Economie [59]. Productivity amounts were inflated to 2020 Euros using the CPI general index [59]. Daily productivity costs for caregivers are based on the average gross annual salary in Belgium in 2020 divided by the total annual days worked.
15-19 y	€31,525	€86.37	
20-24 y	€35,011	€95.92	
25-29 y	€40,673	€111.43	
30-34 y	€46,378	€127.06	
35-39 y	€51,015	€139.77	
40-44 y	€54,856	€150.29	
45-49 y	€56,194	€153.96	
50-54 y	€57,716	€158.13	
55-59 y	€61,813	€169.35	
≥60 y	€67,248	€184.24	

CPI = consumer price index.

[a] Annual productivity estimates were used to calculate indirect costs (discounted lifetime productivity loss) of disease-related mortality (Table A-20).

[b] Daily productivity estimates were used to calculate indirect costs of time spent with disease cases and caregiver time for vaccination.

Table A-20. Age-Specific Life Expectancy and Calculated Lifetime Productivity Lost from Premature Death

Age	Probability of all-cause mortality[a]	Remaining life expectancy (years)[b]	Discounted lifetime productivity lost for death at specific age[c]
1 year	0.000131	80.3	€911,906
10 years	0.000091	71.3	€1,190,895
20 years	0.000384	61.5	€1,430,529
30 years	0.000528	51.7	€1,485,002
40 years	0.001032	42.0	€1,434,974
50 years	0.002504	32.6	€1,299,422
60 years	0.006814	23.8	€1,100,599
70 years	0.016115	15.9	€806,870
80 years	0.045260	9.0	€491,059
90 years	0.152538	4.1	€224,899
100 years[d]	1.000000	0.5	€0

[a] Statbel and SPF Economie [2].

[b] Calculated based on probability of all-cause mortality data [2].

[c] Calculated using age-specific data (by 1-year intervals, not all shown here) and discounted with an annual rate of 3%.

[d] Calculations for death truncated at age 100.

1.1 Appendix References

1. Agentschap Zorg en Gezondheid. Vlaamse overheid van Belgie (Health and Care agency, Flemish Regional Government of Belgium) Vaccinatie Difterie (Diphteria Vaccination). (2013). https://www.zorg-en-gezondheid.be/sites/default/files/2022-12/Difterie_2022.pdf [Accessed 28 October 2021].
2. Statbel, SPF Economie. Belgium National Office for Statistics. Population par lieu de résidence, nationalité, état civil, âge et sexe. (2021). https://statbel.fgov.be/fr/themes/population/structure-de-la-population#figures [Accessed October 5, 2021].
3. ECDC. European Centre for Disease Prevention and Control. Diphtheria: annual epidemiological report for 2017. (2019). https://www.ecdc.europa.eu/sites/default/files/documents/diphtheria-annual-epidemiological-report-2017.pdf [Accessed October 28, 2021].
4. ECDC. European Centre for Disease Prevention and Control. Hepatitis B: annual epidemiological report for 2018. (2020). https://www.ecdc.europa.eu/sites/default/files/documents/HEPB_AER_2018_Report.pdf [Accessed October 28, 2021].
5. Reinert P, Liwartowski A, Dabernat H, Guyot C, Boucher J, Carrere C. Epidemiology of Haemophilus influenzae type b disease in France. Vaccine (1993) 11 Suppl 1:S38-42. doi: 10.1016/0264-410x(93)90158-t
6. ECDC. European Centre for Disease Prevention and Control. Haemophilus influenzae, annual epidemiological report for 2017. (2019). https://www.ecdc.europa.eu/sites/default/files/documents/haemophilus-influenzae-annual-epidemiological-report-2017.pdf [Accessed October 28, 2021].
7. Van Casteren V. Epidemiology of measles and mumps in Belgium, 1994-1995. Arch Public Health (1997) 15.
8. ECDC. European Centre for Disease Prevention and Control. Measles, annual epidemiological report for 2019. (2020). https://www.ecdc.europa.eu/sites/default/files/documents/measles-2019-aer.pdf [Accessed October 28, 2021].
9. Noah N. Surveillance of bacterial meningitis in Europe 1999/2000. Euro Surveill (2002) 6:ii-2116. doi: 10.2807/esw.06.15.02116-en
10. ECDC. European Centre for Disease Prevention and Control. Survaillance of invasive bacterial diseases in Europe 2007. (2010). https://www.ecdc.europa.eu/sites/default/files/media/en/publications/Publications/101011_SUR_Surveillance_of_invasive_bacterial_diseases_in_Europe_2007.pdf [Accessed October 28, 2021].
11. Jacquinet S, Mattheus W, Lajot A, Wyndham-Thomas C. Institut Scientifique de Santé Publique. Surveillance épidémiologique des infections invasives à méningocoques - 2018. (2018).
12. ECDC. European Centre for Disease Prevention and Control. Mumps, annual epidemiological report for 2017. (2020). https://www.ecdc.europa.eu/sites/default/files/documents/mumps-2017-aer.pdf [Accessed October 28, 2021].
13. Gałązka A. Czy możemy lepiej zapobiegać krztuścowi? I. Zmiany w epidemiologii krztuśca. Przeg Epid (1997) 51:275-84.
14. ECDC. European Centre for Disease Prevention and Control. Pertussis: annual epidemiological report for 2018. (2020). https://www.ecdc.europa.eu/sites/default/files/documents/AER_for_2018_pertussis.pdf [Accessed October 28, 2021].
15. Cockburn WC, Drozdov SG. Poliomyelitis in the world. Bull World Health Organ (1970) 42:405-17.
16. ECDC. European Centre for Disease Prevention and Control. Poliomyelitis, annual epidemiological report for 2017. (2019). https://www.ecdc.europa.eu/en/publications-data/poliomyelitis-annual-epidemiological-report-2017 [Accessed October 28, 2021].
17. Mendes da Costa E, Grammens T, Litzroth A, Maes V, Muyldermans G, Quoiln S, et al. Institut Scientifique de Santé Publique. Maladies Infectieuses pédiatriques a prévention vaccinale. Rapport annuel, 2015. (2015).
18. Blommaert A, Hanquet G, Willem L, Theeten H, Thiry N, Bilcke J, et al. Belgian Health Care Knowledge Centre (KCE). Use of pneumococcal vaccines in the elderly: an economic evaluation. Health technology assessment (HTA). (2016). Report No.: KCE Reports 274. D/2016/10.273/79.
19. Vergison A, Tuerlinckx D, Verhaegen J, Malfroot A. Epidemiologic features of invasive pneumococcal disease in Belgian children: passive surveillance is not enough. Pediatrics (2006) 118:e801-9. doi: 10.1542/peds.2005-3195

20. Braeye T, Wyndham-Thomas C, Lagrou K, Grammens T, Desmet S. Institut Scientifique de Santé Publique. Epidemiologische surveillance van invasieve pneumokokkeninfecties (IPD). (2018).
21. Beutels P, Van Damme P, Oosterhuis-Kafeja F. Belgian Health Care Knowledge Centre (KCE). Effects and costs of pneumococcal conjugate vaccination of Belgian children. Health technology assessment (HTA). (2006). Report No.: KCE reports 33C (D/2006/10.273/53).
22. Beutels P, Blommaert A, Hanquet G, Bilcke J, Thiry N, Sabbe M, et al. Belgian Health Care Knowledge Centre (KCE). Cost-effectiveness of 10- and 13-valent pneumococcal conjugate vaccines in childhood. Health technology assessment (HTA). (2011). Report No.: 155C. D/2011/10.273/21.
23. Kawai K, Adil EA, Barrett D, Manganella J, Kenna MA. Ambulatory Visits for otitis media before and after the introduction of pneumococcal conjugate vaccination. J Pediatr (2018) 201:122-7 e1. doi: 10.1016/j.jpeds.2018.05.047
24. Jit M, Bilcke J, Mangen MJ, Salo H, Melliez H, Edmunds WJ, et al. The cost-effectiveness of rotavirus vaccination: comparative analyses for five European countries and transferability in Europe. Vaccine (2009) 27:6121-8. doi: 10.1016/j.vaccine.2009.08.030
25. Bilcke J, Van Damme P, Beutels P. Cost-effectiveness of rotavirus vaccination: exploring caregiver(s) and "no medical care" disease impact in Belgium. Med Decis Making (2009) 29:33-50. doi: 10.1177/0272989x08324955
26. Bilcke J, Van Damme P, De Smet F, Hanquet G, Van Ranst M, Beutels P. The health and economic burden of rotavirus disease in Belgium. Eur J Pediatr (2008) 167:1409-19. doi: 10.1007/s00431-008-0684-3
27. Sabbe M, Berger N, Blommaert A, Ogunjimi B, Grammens T, Callens M, et al. Sustained low rotavirus activity and hospitalisation rates in the post-vaccination era in Belgium, 2007 to 2014. Euro Surveill (2016) 21. doi: 10.2807/1560-7917.ES.2016.21.27.30273
28. Zeller M, Rahman M, Heylen E, De Coster S, De Vos S, Arijs I, et al. Rotavirus incidence and genotype distribution before and after national rotavirus vaccine introduction in Belgium. Vaccine (2010) 28:7507-13. doi: 10.1016/j.vaccine.2010.09.004
29. NIZP-PZH. Pan´stwowy Zakład Higieny. Choroby zakaz´ne i zatrucia w Polsce w 1999 roku. (1999). http://wwwold.pzh.gov.pl/oldpage/epimeld/1999/Ch_1999.pdf [Accessed August 5, 2020].
30. NIZP-PZH. Pan´stwowy Zakład Higieny. Choroby zakaz´ne i zatrucia w Polsce w 2000 roku. (2000). http://wwwold.pzh.gov.pl/oldpage/epimeld/2000/Ch_2000.pdf [Accessed August 5, 2020].
31. NIZP-PZH. Pan´stwowy Zakład Higieny. Choroby zakaz´ne i zatrucia w Polsce w 2001 roku. (2001). http://wwwold.pzh.gov.pl/oldpage/epimeld/2001/Ch_2001.pdf [Accessed August 5, 2020].
32. NIZP-PZH. Pan´stwowy Zakład Higieny. Choroby zakaz´ne i zatrucia w Polsce w 2002 roku. (2002). http://wwwold.pzh.gov.pl/oldpage/epimeld/2002/Ch_2002.pdf [Accessed August 5, 2020].
33. NIZP-PZH. Pan´stwowy Zakład Higieny. Choroby zakaz´ne i zatrucia w Polsce w 2002 roku. (2003). http://wwwold.pzh.gov.pl/oldpage/epimeld/2003/Ch_2003.pdf [Accessed August 5, 2020].
34. ECDC. European Centre for Disease Prevention and Control. Measles and rubella surveillance report, 2017. (2018). https://www.ecdc.europa.eu/sites/default/files/documents/Measles-and-Rubella-Surveillance-2017.pdf [Accessed October 28, 2021].
35. Kostrzewski J, editor. Choroby zakaźne w Polsce i ich zwalczanie w latach (PZWL) 1919 -1962. Warszawa: Państwowy Zakład Wydawnictwa Lekarskich; (1964).
36. ECDC. European Centre for Disease Prevention and Control. Tetanus: annual epidemiological report for 2017. (2019). https://www.ecdc.europa.eu/sites/default/files/documents/tetanus-annual-epidemiological-report-2017_0.pdf [Accessed October 28, 2021].
37. Zhou F, Reef S, Massoudi M, Papania MJ, Yusuf HR, Bardenheier B, et al. An economic analysis of the current universal 2-dose measles-mumps-rubella vaccination program in the United States. J Infect Dis (2004) 189 Suppl 1:S131-45. doi: 10.1086/378987
38. Zhou F, Shefer A, Wenger J, Messonnier M, Wang LY, Lopez A, et al. Economic evaluation of the routine childhood immunization program in the United States, 2009. Pediatrics (2014) 133:577-85. doi: 10.1542/peds.2013-0698
39. Widdowson MA, Meltzer MI, Zhang X, Bresee JS, Parashar UD, Glass RI. Cost-effectiveness and potential impact of rotavirus vaccination in the United States. Pediatrics (2007) 119:684-97. doi: 10.1542/peds.2006-2876

40. Bilcke J, Beutels P, De Smet F, Hanquet G, Van Ranst M, Van Damme P. Cost-effectiveness analysis of rotavirus vaccination of Belgian infants. Health technology assessment (HTA). Brussels: Belgian Health Care Knowledge Centre (KCE). (2007). Report No.: KCE reports 54C, D2007/10.273/11.
41. Hanquet G, Christensen H, Agnew E, Trotter C, Robays J, Dubois C, et al. Belgian Health Care Knowledge Centre (KCE). A quadrivalent vaccine against serogroup B meningococcal disease: a cost-effectiveness study. Health technology assessment (HTA). (2014). Report No.: KCE Reports 231. D/2014/10.273/77.
42. McNeil MM, Weintraub ES, Duffy J, Sukumaran L, Jacobsen SJ, Klein NP, et al. Risk of anaphylaxis after vaccination in children and adults. J Allergy Clin Immunol (2016) 137:868-78. doi: 10.1016/j.jaci.2015.07.048
43. Erlewyn-Lajeunesse M, Hunt LP, Heath PT, Finn A. Anaphylaxis as an adverse event following immunisation in the UK and Ireland. Arch Dis Child (2012) 97:487-90. doi: 10.1136/archdischild-2011-301163
44. Bohlke K, Davis RL, Marcy SM, Braun MM, DeStefano F, Black SB, et al. Risk of anaphylaxis after vaccination of children and adolescents. Pediatrics (2003) 112:815-20. doi: 10.1542/peds.112.4.815
45. Zhou F, Santoli J, Messonnier ML, Yusuf HR, Shefer A, Chu SY, et al. Economic evaluation of the 7-vaccine routine childhood immunization schedule in the United States, 2001. Arch Pediatr Adolesc Med (2005) 159:1136-44. doi: 10.1001/archpedi.159.12.1136
46. Kamiya H, Cho BH, Messonnier ML, Clark TA, Liang JL. Impact and cost-effectiveness of a second tetanus toxoid, reduced diphtheria toxoid, and acellular pertussis (Tdap) vaccine dose to prevent pertussis in the United States. Vaccine (2016) 34:1832-8. doi: 10.1016/j.vaccine.2016.02.027
47. Lee GM, Salomon JA, LeBaron CW, Lieu TA. Health-state valuations for pertussis: methods for valuing short-term health states. Health Qual Life Outcomes (2005) 3:17. doi: 10.1186/1477-7525-3-17
48. Tu HA, Deeks SL, Morris SK, Strifler L, Crowcroft N, Jamieson FB, et al. Economic evaluation of meningococcal serogroup B childhood vaccination in Ontario, Canada. Vaccine (2014) 32:5436-46. doi: 10.1016/j.vaccine.2014.07.096
49. Rubin JL, McGarry LJ, Strutton DR, Klugman KP, Pelton SI, Gilmore KE, et al. Public health and economic impact of the 13-valent pneumococcal conjugate vaccine (PCV13) in the United States. Vaccine (2010) 28:7634-43. doi: 10.1016/j.vaccine.2010.09.049
50. Anyiwe K, Richardson M, Brophy J, Sander B. Assessing adolescent immunization options for pertussis in Canada: A cost-utility analysis. Vaccine (2020) 38:1825-33. doi: 10.1016/j.vaccine.2019.12.021
51. Philipson TJ, Thornton Snider J, Chit A, Green S, Hosbach P, Tinkham Schwartz T, et al. The social value of childhood vaccination in the United States. Am J Manag Care (2017) 23:41-7.
52. Fowler A, Stodberg T, Eriksson M, Wickstrom R. Long-term outcomes of acute encephalitis in childhood. Pediatrics (2010) 126:e828-35. doi: 10.1542/peds.2009-3188
53. Martin A, Batty A, Roberts JA, Standaert B. Cost-effectiveness of infant vaccination with RIX4414 (Rotarix) in the UK. Vaccine (2009) 27:4520-8. doi: 10.1016/j.vaccine.2009.05.006
54. Cleemput I, Neyt M, Van de Sande S, Thiry N. Belgian Health Care Knowledge Centre (KCE). Belgian guidelines for economic evaluations and budget impact analyses: second edition. Health technology assessment (HTA). (2012). Report No.: KCE Report 183C. D/2012/10.273/54.
55. RIZIV/INAMI. Institut National d'Assurance Maladie-Invalidité. Programme web - Médicaments. (2021). https://ondpanon.riziv.fgov.be/SSPWebApplicationPublic/fr/Public/ProductSearch [Accessed July 30, 2021].
56. Carrico J, La EM, Talbird SE, Chen YT, Nyaku MK, Carias C, et al. Value of the immunization program for children in the 2017 US birth cohort. Pediatrics (2022). doi: 10.1542/peds.2021-056013
57. Ekwueme DU, Strebel PM, Hadler SC, Meltzer MI, Allen JW, Livengood JR. Economic evaluation of use of diphtheria, tetanus, and acellular pertussis vaccine or diphtheria, tetanus, and whole-cell pertussis vaccine in the United States, 1997. Arch Pediatr Adolesc Med (2000) 154:797-803. doi: 10.1001/archpedi.154.8.797
58. Whitney CG, Zhou F, Singleton J, Schuchat A, Centers for Disease Control and Prevention. Benefits from immunization during the vaccines for children program era - United States, 1994-2013. MMWR Morb Mortal Wkly Rep (2014) 63:352-5.
59. Statbel, SPF Economie. Belgium National Office for Statistics. Indice des prix à la consommation (CPI). (2021). https://statbel.fgov.be/fr/themes/prix-la-consommation/indice-des-prix-la-consommation#figures [Accessed October 28, 2021].
60. Margolis HS, Coleman PJ, Brown RE, Mast EE, Sheingold SH, Arevalo JA. Prevention of hepatitis B virus transmission by immunization. An economic analysis of current recommendations. JAMA (1995) 274:1201-8.

61. Tormans G, Carrin G, Clara R, Eyelenbosch W, van Damme P. University of Antwerp, Faculty of Business and Economics. Cost-effectiveness analysis of prenatal screening and vaccination against hepatitis B virus - the case of Belgium. SESO working papers. (1990).

62. Miriti MK, Billah K, Weinbaum C, Subiadur J, Zimmerman R, Murray P, et al. Economic benefits of hepatitis B vaccination at sexually transmitted disease clinics in the U.S. Public Health Rep (2008) 123:504-13. doi: 10.1177/003335490812300412

63. CDC. Centers for Disease and Control and Prevention. Hepatitis B questions and answers for health professionals. (2018). https://www.cdc.gov/hepatitis/hbv/hbvfaq.htm [Accessed April 23, 2019].

64. Gerkens S, Thiry N, Hulsaert F, Robays J. Belgian Health Care Knowledge Centre (KCE). Economic evaluation of novel direct acting antiviral (DAA) treatment strategies for chronic hepatitis C. Health Technology Assessment (HTA). (2016). Report No.: KCE Reports 276. D/2016/10.273/88.

65. Mulley AG, Silverstein MD, Dienstag JL. Indications for use of hepatitis B vaccine, based on cost-effectiveness analysis. N Engl J Med (1982) 307:644-52. doi: 10.1056/NEJM198209093071103

66. Bloom BS, Hillman AL, Fendrick AM, Schwartz JS. A reappraisal of hepatitis B virus vaccination strategies using cost-effectiveness analysis. Ann Intern Med (1993) 118:298-306. doi: 10.7326/0003-4819-118-4-199302150-00009

67. Arevalo JA, Washington AE. Cost-effectiveness of prenatal screening and immunization for hepatitis B virus. JAMA (1988) 259:365-9.

68. Krahn M, Detsky AS. Should Canada and the United States universally vaccinate infants against hepatitis B? A cost-effectiveness analysis. Med Decis Making (1993) 13:4-20. doi: 10.1177/0272989X9301300103

69. Dhankhar P, Nwankwo C, Pillsbury M, Lauschke A, Goveia MG, Acosta CJ, et al. Public health impact and cost-effectiveness of hepatitis A vaccination in the United States: a disease transmission dynamic modeling approach. Value Health (2015) 18:358-67. doi: 10.1016/j.jval.2015.02.004

70. Wolfe RA, Merion RM, Roys EC, Port FK. Trends in organ donation and transplantation in the United States, 1998-2007. Am J Transplant (2009) 9:869-78. doi: 10.1111/j.1600-6143.2009.02564.x

71. US Bureau of Labor Statistics. Consumer Price Index - All urban consumers: all items [CUSR0000SA0]. (2019). https://www.bls.gov/cpi/data.htm [Accessed March 25, 2019].

72. Zhou F, Bisgard KM, Yusuf HR, Deuson RR, Bath SK, Murphy TV. Impact of universal Haemophilus influenzae type b vaccination starting at 2 months of age in the United States: an economic analysis. Pediatrics (2002) 110:653-61. doi: 10.1542/peds.110.4.653

73. Phoenix G, Das S, Joshi M. Diagnosis and management of cellulitis. BMJ (2012) 345:e4955. doi: 10.1136/bmj.e4955

74. Garpenholt O, Silfverdal SA, Levin LA. Economic evaluation of general childhood vaccination against Haemophilus influenzae type b in Sweden. Scand J Infect Dis (1998) 30:5-10. doi: 10.1080/003655498750002222

75. Christie D, Rashid H, El-Bashir H, Sweeney F, Shore T, Booy R, et al. Impact of meningitis on intelligence and development: a systematic review and meta-analysis. PLoS One (2017) 12:e0175024. doi: 10.1371/journal.pone.0175024

76. Jit M. The risk of sequelae due to pneumococcal meningitis in high-income countries: a systematic review and meta-analysis. J Infect (2010) 61:114-24. doi: 10.1016/j.jinf.2010.04.008

77. Cochi SL, Broome CV, Hightower AW. Immunization of US children with Hemophilus influenzae type b polysaccharide vaccine. A cost-effectiveness model of strategy assessment. JAMA (1985) 253:521-9.

78. Beutels P, Van Damme P, Van Casteren V, Gay NJ, De Schrijver K, Meheus A. The difficult quest for data on "vanishing" vaccine-preventable infections in Europe: the case of measles in Flanders (Belgium). Vaccine (2002) 20:3551-9. doi: 10.1016/s0264-410x(02)00335-3

79. Pelletier L, Chung P, Duclos P, Manga P, Scott J. A benefit-cost analysis of two-dose measles immunization in Canada. Vaccine (1998) 16:989-96. doi: 10.1016/s0264-410x(97)00281-8

80. Sciensano. Surveillance épidémiologique des oreillons (Mumps surveilance), 2018. (2018). https://epidemio.wiv-isp.be/ID/reports/Oreillons_Epid%C3%A9miologie_2018_fr_FIN.pdf [Accessed October 28, 2021].

81. de Greeff SC, Lugnér AK, van den Heuvel DM, Mooi FR, de Melker HE. Economic analysis of pertussis illness in the Dutch population: implications for current and future vaccination strategies. Vaccine (2009) 27:1932-7. doi: 10.1016/j.vaccine.2009.01.106

82. CDC. Centers for Disease and Control and Prevention. Pertussis (whooping cough): surveillance and reporting. (2019). https://www.cdc.gov/pertussis/surv-reporting.html [Accessed June 5, 2020].

83. Willem L, Blommaert A, Hanquet G, Thiry N, Bilcke J, Theeten H, et al. Economic evaluation of pneumococcal vaccines for adults aged over 50 years in Belgium. Hum Vaccin Immunother (2018) 14:1218-29. doi: 10.1080/21645515.2018.1428507

84. Roush SW, Murphy TV. Historical comparisons of morbidity and mortality for vaccine-preventable diseases in the United States. JAMA (2007) 298:2155-63. doi: 10.1001/jama.298.18.2155

85. CDC. Impact of vaccines universally recommended for children--United States, 1990-1998. MMWR Morb Mortal Wkly Rep (1999) 48:243-8.

86. Chan B, McIntyre A, Mittmann N, Teasell R, Wolfe DL. Economic evaluation of spinal cord injury. In: Eng JJ, Teasell RW, Miller WC, Wolfe DL, Townson AF, Hsieh JTC, et al., editors. Spinal cord injury rehabilitation evidence, version 5. 2014. p. 1-21.

87. CDC. Epidemiology and prevention of vaccine-preventable diseases. Hamborsky J, Kroger A, Wolfe S, editors. Washington, DC: Public Health Foundation; (2015).

88. WHO. World Health Organization. Poliomyelitis. (2019). https://www.who.int/en/news-room/fact-sheets/detail/poliomyelitis [Accessed April 30, 2019].

89. CDC. Centers for Disease Control and Prevention. 2017-2018 estimated influenza illnesses, medical visits, hospitalizations, and deaths and estimated influenza illnesses, medical visits, hospitalizations, and deaths averted by vaccination in the United States. (2019). https://www.cdc.gov/flu/about/burden-averted/2017-2018.htm [Accessed May 16, 2020].

90. CDC. Centers for Disease and Control and Prevention. National Notifiable Diseases Surveillance System. 2017 annual tables of infectious diseases data. (2018). https://www.cdc.gov/nndss/infectious-tables.html [Accessed April 2, 2019].

91. CDC. Centers for Disease and Control and Prevention. National Notifiable Diseases Surveillance System. 2016 annual tables of infectious diseases data. (2017). https://wwwn.cdc.gov/nndss/infectious-tables.html [Accessed May 1, 2020].

92. Adams DA, Thomas KR, Jajosky RA, Foster L, Baroi G, Sharp P, et al. Summary of Notifiable Infectious Diseases and Conditions - United States, 2015. MMWR Morb Mortal Wkly Rep (2017) 64:1-143. doi: 10.15585/mmwr.mm6453a1

93. Adams DA, Thomas KR, Jajosky RA, Foster L, Sharp P, Onweh DH, et al. Summary of Notifiable Infectious Diseases and Conditions - United States, 2014. MMWR Morb Mortal Wkly Rep (2016) 63:1-152. doi: 10.15585/mmwr.mm6354a1

94. Lugner AK, Mollema L, Ruijs WL, Hahne SJ. A cost-utility analysis of antenatal screening to prevent congenital rubella syndrome. Epidemiol Infect (2010) 138:1172-84. doi: 10.1017/S0950268809991336

95. Hatziandreu E, Brown RE, Halpern MT. Battelle Inc. A cost benefit analysis of the measles-mumps-rubella (MMR) vaccine: report prepared for the Centers for Disease Control and Prevention. (1994).

96. WHO. World Health Organization. Global burden of disease 2004 update: disability weights for diseases and conditions. (2004).

97. CDC. Centers for Disease Control and Prevention. Manual for the surveillance of vaccine-preventable diseases. (2012).

98. Chahal HS, Peters MG, Harris AM, McCabe D, Volberding P, Kahn JG. Cost-effectiveness of hepatitis B virus infection screening and treatment or vaccination in 6 high-risk populations in the United States. Open Forum Infect Dis (2019) 6:ofy353. doi: 10.1093/ofid/ofy353

99. CDC. Centers for Disease Control and Prevention. Haemophilus influenzae disease (including Hib): diagnosis and treatment. (2014).

100. Oostenbrink R, HA AM, Essink-Bot ML. The EQ-5D and the Health Utilities Index for permanent sequelae after meningitis: a head-to-head comparison. J Clin Epidemiol (2002) 55:791-9. doi: 10.1016/s0895-4356(02)00448-1

101. National Health Service. Mumps. (2015).

102. Greer AL, Fisman DN. Use of models to identify cost-effective interventions: pertussis vaccination for pediatric health care workers. Pediatrics (2011) 128:e591-9. doi: 10.1542/peds.2010-0796

103. Bennett JE, Sumner W, 2nd, Downs SM, Jaffe DM. Parents' utilities for outcomes of occult bacteremia. Arch Pediatr Adolesc Med (2000) 154:43-8.

104. Oh PI, Maerov P, Pritchard D, Knowles SR, Einarson TR, Shear NH. A cost-utility analysis of second-line antibiotics in the treatment of acute otitis media in children. Clin Ther (1996) 18:160-82. doi: 10.1016/s0149-2918(96)80188-3

105. Khan MM, Ehreth J. Costs and benefits of polio eradication: a long-run global perspective. Vaccine (2003) 21:702-5. doi: 10.1016/s0264-410x(02)00584-4

106. Harvard Medical School. Polio: what is it? (2019). https://www.health.harvard.edu/a_to_z/polio-a-to-z#:~:text=The%20fever%20and%20other%20symptoms,can%20continue%20for%20two%20years [Accessed June 30, 2020].

107. Prosser LA, Bridges CB, Uyeki TM, Hinrichsen VL, Meltzer MI, Molinari NA, et al. Health benefits, risks, and cost-effectiveness of influenza vaccination of children. Emerg Infect Dis (2006) 12:1548. doi: 10.3201/eid1210.051015

108. Martin A, Cottrell S, Standaert B. Estimating utility scores in young children with acute rotavirus gastroenteritis in the UK. J Med Econ (2008) 11:471-84. doi: 10.3111/13696990802321047

109. National Health Service. Rubella. (2015).

110. Berge JJ, Drennan DP, Jacobs RJ, Jakins A, Meyerhoff AS, Stubblefield W, et al. The cost of hepatitis A infections in American adolescents and adults in 1997. Hepatology (2000) 31:469-73. doi: 10.1002/hep.510310229

111. Lieu TA, Ray GT, Black SB, Butler JC, Klein JO, Breiman RF, et al. Projected cost-effectiveness of pneumococcal conjugate vaccination of healthy infants and young children. JAMA (2000) 283:1460-8. doi: 10.1001/jama.283.11.1460

112. Ray GT, Whitney CG, Fireman BH, Ciuryla V, Black SB. Cost-effectiveness of pneumococcal conjugate vaccine: evidence from the first 5 years of use in the United States incorporating herd effects. Pediatr Infect Dis J (2006) 25:494-501. doi: 10.1097/01.inf.0000222403.42974.8b

113. CDC. Centers for Disease and Control and Prevention. What is polio? (2019). https://www.cdc.gov/polio/what-is-polio/index.htm [Accessed June 30, 2020].

114. Mast TC, Walter EB, Bulotsky M, Khawaja SS, DiStefano DJ, Sandquist MK, et al. Burden of childhood rotavirus disease on health systems in the United States. Pediatr Infect Dis J (2010) 29:e19-25. doi: 10.1097/inf.0b013e3181ca7e2e
